# Perspectives for Using CO_2_ as a Feedstock for Biomanufacturing of Fuels and Chemicals

**DOI:** 10.3390/bioengineering10121357

**Published:** 2023-11-26

**Authors:** Elif Kurt, Jiansong Qin, Alexandria Williams, Youbo Zhao, Dongming Xie

**Affiliations:** 1 Department of Chemical Engineering, University of Massachusetts, Lowell, MA 01854, USA; elif_kurt@student.uml.edu (E.K.); jiansong_qin@student.uml.edu (J.Q.); alexandria_williams@student.uml.edu (A.W.); 2 Physical Sciences Inc., 20 New England Business Ctr., Andover, MA 01810, USA; yzhao@psicorp.com

**Keywords:** metabolic engineering, CO_2_ fixation, feedstock, biomanufacturing, electrochemical catalysis, microbial electrosynthesis

## Abstract

Microbial cell factories offer an eco-friendly alternative for transforming raw materials into commercially valuable products because of their reduced carbon impact compared to conventional industrial procedures. These systems often depend on lignocellulosic feedstocks, mainly pentose and hexose sugars. One major hurdle when utilizing these sugars, especially glucose, is balancing carbon allocation to satisfy energy, cofactor, and other essential component needs for cellular proliferation while maintaining a robust yield. Nearly half or more of this carbon is inevitably lost as CO_2_ during the biosynthesis of regular metabolic necessities. This loss lowers the production yield and compromises the benefit of reducing greenhouse gas emissions—a fundamental advantage of biomanufacturing. This review paper posits the perspectives of using CO_2_ from the atmosphere, industrial wastes, or the exhausted gases generated in microbial fermentation as a feedstock for biomanufacturing. Achieving the carbon-neutral or -negative goals is addressed under two main strategies. The one-step strategy uses novel metabolic pathway design and engineering approaches to directly fix the CO_2_ toward the synthesis of the desired products. Due to the limitation of the yield and efficiency in one-step fixation, the two-step strategy aims to integrate firstly the electrochemical conversion of the exhausted CO_2_ into C_1_/C_2_ products such as formate, methanol, acetate, and ethanol, and a second fermentation process to utilize the CO_2_-derived C_1_/C_2_ chemicals or co-utilize C_5_/C_6_ sugars and C_1_/C_2_ chemicals for product formation. The potential and challenges of using CO_2_ as a feedstock for future biomanufacturing of fuels and chemicals are also discussed.

## 1. Introduction

Carbon emission to our ecosystem and its accumulation in its highly oxidized state, carbon dioxide (CO_2_), are the primary contributing factors to global climate change [1]. Since the 1960s, the total CO_2_ emissions have rapidly increased, with a net annual escalation rate of 2.11% in recent years [2]. The push for carbon neutrality necessitates reimagining our feedstock sources. Over 90% of our chemicals and fuels are manufactured from fossil feedstocks, driving the need to transition toward a more circular industry model. G20 economies have implemented carbon emission taxes ranging from $3 to $60 per ton to incentivize CO_2_ capture from industrial processes [3]. The cost of carbon capture varies based on the CO_2_ source [4]. This suggests that, in some countries, obtaining CO_2_ at zero cost may be possible. Therefore, exploring the potential of capturing and utilizing CO_2_ is essential to mitigate the global warming challenge.

Photosynthesis is the natural way to capture CO_2_ from the atmosphere and fix it into sugars or carbohydrates, which can then be used as the feedstocks for microbial cells to produce fuels and chemicals by green plants and algae. Therefore, biomanufacturing is considered more sustainable than chemical manufacturing with petroleum-based feedstocks. However, the production of biomass through the photosynthesis process still suffers the challenge of high-cost processing and low-energy efficiency [5]. While photosynthesis is a marvel of nature, its energy efficiency seldom surpasses 3%, constraining its industrial applicability. Moreover, using agricultural crops to provide feedstocks for biomanufacturing poses a sustainability challenge as it hinders food production and threatens biodiversity when natural areas are used for agricultural purposes.

Sugars such as glucose are the most widely used substrate for biomanufacturing in laboratory and industrial settings for historical and practical reasons. However, employing glucose may repress gene expression and specific biosynthetic pathways for certain biomanufacturing products. In most cases, glucose may also cause several limitations in cell metabolism, resulting in carbon loss as CO_2_ [6]. This is particularly noticeable when the product of interest requires long synthetic routes from the starting carbon source when it has chemical properties distinct from the substrate or when unfavorable substrates are used, ultimately leading to low product yield [7].

Despite the predominant dependence of current industrial biomanufacturing processes on carbon-intensive carbohydrate substrates, including the C_5_/C_6_ sugars such as xylose and glucose derived from cellulosic biomass, it is worth acknowledging that the feedstock and raw materials significantly contribute to the overall cost of biomanufacturing [8]. Reducing the cost can be achieved by using more economical raw materials and designing new microbial cell factories that can efficiently utilize alternative feedstocks. Some microorganisms exhibit the inherent capability or possess the potential to metabolize C_1_ and C_2_ substrates [9]. These C_1_ substrates, comprising CO_2_, carbon monoxide (CO), methane (CH_4_), methanol (CH_3_OH), and formate (CHOO^−^) [10], and C_2_ substrates, comprising mainly ethanol and acetate [11], hold the gains of being inexpensive, naturally abundant, and straightforward manufacturing along with their abundant availability as by-products and industrial wastes [9]. Owing to the worldwide attention to continuous conversion of greenhouse gases, specifically CO_2_ [12] to recover its diminished economic worth, scientists have a special interest in designing innovative CO_2_ fixation methods with microbial entities, thereby assisting them in the synthesis of crucial substrate precursors (C_1_ and C_2_ chemicals) having the inherent capability to serve as biomanufacturing substrate in numerous processes [13,14].

However, the utilization of CO_2_-derived C_1_/C_2_ chemicals for biomanufacturing is challenged by the inefficiency of conversion into desired bioproducts by native microorganisms, resulting in relatively lower productivity, limited energy availability, and deprived carbon yield, as compared with the utilization of C_5_/C_6_ sugars [14]. To address the associated challenges, major efforts have been made in the field of synthetic biology and metabolic engineering to evolve both natural microbes [15] and/or heterologous microorganisms by engineering the pathways or enzymes to improve their C_1_ and C_2_ substrate-utilizing capabilities [14,16,17,18,19]. Such interventions may range from enhancing native pathways to integrating entirely novel ones crafted from a deep understanding of metabolic networks and enzymology to improve carbon-fixation efficiency [19].

Furthermore, as we delve into microbial fermentation for carbon fixation, we stumble upon its nuanced challenges. One of the pivotal concerns is the significant carbon loss, especially in the format of CO_2_ during microbial fermentation [20,21], which comprises the advantageous of using biomanufacturing as one of the major efforts in reducing greenhouse gas emission [22]. Therefore, recycling the exhausted CO_2_ back to the microbial fermentation process is also critical to the success of biomanufacturing.

**Figure 1:**
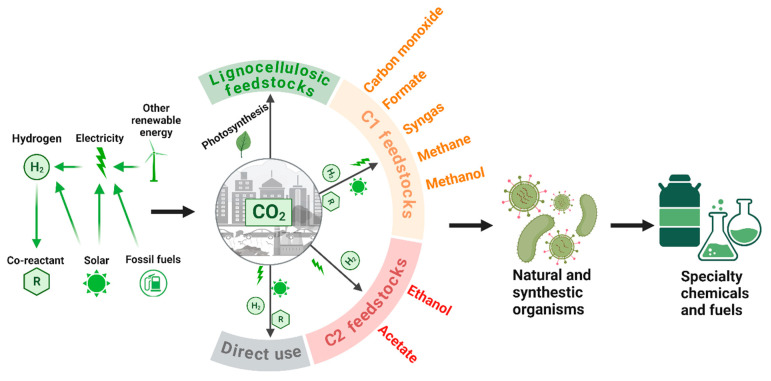
An overview of the CO_2_ conversion approaches and using CO_2_-derived C_1_/C_2_ chemicals for biomanufacturing of common products. Energy conversion and sources used in the conversion are summarized on the left. After CO_2_ is converted from inorganic to organic carbon substrates, various valuable chemicals can be biomanufactured through natural and synthetic microorganisms. The figure was generated using Biorender.

This review aims to investigate the perspectives for using CO_2_ as a feedstock for biomanufacturing. First, the one-step strategy is discussed, which uses novel metabolic pathway design in microbes and engineering approaches to directly fix CO_2_ and convert it into desired fermentation products. Due to the limitation of the efficiency of one-step CO_2_ fixation, we further discuss the two-step strategy, which aims to integrate a first electrochemical fixation of CO_2_ into C_1_/C_2_ products such as formate, methanol, acetate, and ethanol and a second fermentation unit co-fed with the original C_5_/C_6_ sugars and the CO_2_-derived C_1_/C_2_ chemicals. The great potentials and challenges of using CO_2_ as a feedstock for future biomanufacturing of various fermentation products are discussed. Figure 1 shows an overview of the CO_2_ conversion approaches and the uses of CO_2_-derived C_1_/C_2_ chemicals for biomanufacturing of common products is shown.

## 2. State of the Art of Current Technologies

The conversion of CO_2_ into value-added chemicals using microbes as biocatalysts is an exciting field of research with the potential to revolutionize biomanufacturing processes [23]. For using CO_2_ as the feedstock for biomanufacturing, both one-step and two-step strategies can be applied. Table 1 summarizes the general strategies for fixation of CO_2_ for biomanufacturing. The one-step strategy uses the native or engineered pathways to directly fix CO_2_ and convert it into desired fermentation products, typically with multiple carbons. Since CO_2_ has the lowest energy format, producing high-value chemicals with a higher energy format requires extra energy; this can be achieved by either plants, algae, or cyanobacteria via a photosynthesis process that uses light as the energy source or other microorganisms with cofeeding higher energy-intensive chemicals such as hydrogen gas. The two-step strategy uses a hybrid electrochemical and biochemical conversion approach to fix CO_2_ and convert it to the desired fermentation products at higher yield and efficiency, where the first step uses an electrochemical catalysis process to convert CO_2_ into C_1_/C_2_ chemicals, followed by a second fermentation step to further convert C_1_/C_2_ chemicals into desired products by native or engineered microorganisms. 

### 2.1. One-Step Strategy—Direct Conversion

Internal carbon sequestration has taken many different forms throughout history. Even before the evolution of eukaryotic plants utilizing photosynthesis and light to convert CO_2_ and energy from light to compose simple sugars, single-celled organisms had already developed mechanisms to capture atmospheric CO_2_ and transform it into essential compounds for the cell’s development. These primitive mechanisms, especially those in microorganisms like *Acetogens* and *Methanogens*, have been shown to be highly efficient in, utilizing unique proteins and metabolic pathways for carbon sequestration [1]. Furthermore, microorganisms, especially microalgae and cyanobacteria, exhibit significant advantages over higher plants in their capacity for CO_2_ fixation as they can yield higher solar energy retention and the potential for year-round growth compared to their more complex plant counterparts [24]. While microalgae are well-recognized for their CO_2_ fixation capabilities, bacteria present advantages that cannot be overlooked [25]. Microalgae cultivation can be subject to biocontamination over prolonged use from fungal and bacterial species and often run into issues pertaining to even distribution of sun exposure over larger microalgae ponds due to their preferred growth environments, vastly limiting their ability to be utilized on an industrial scale without major alternations to the water infrastructure the microalgae is grown on. Bacteria and some yeasts, on the other hand, have been widely used in biotechnology industry due to their inherent compatibility to produce chemicals and their rapid growth rates and life cycles. Further, they are more inclined to accept DNA during genetic modification in the form of plasmids and genomic alternations. This ability allows bacteria and yeast to have DNA introduced into their cells of enzymes to complete metabolic pathways previously incompletely represented in the cells and allow production of specialized products, including bioalcohols and essential fatty acids. Through this biotechnological approach, CO_2_ can be directly converted into value-added products, offering an advantage over traditional methods like catalytic conversion, which demand energy-intensive conditions [23]. 

In this section, we will provide an overview of the one-step strategy for directly using CO_2_ as the feedstock for biomanufacturing, which includes (1) natural CO_2_ fixation pathways, (2) synthetic CO_2_ fixation pathways, (3) host selection and reducing power required for biomanufacturing with CO_2_, and (4) using microbial electrosynthesis to utilize CO_2_ for biomanufacturing.

#### 2.1.1. Natural CO_2_ Fixation Pathways

**Figure 2:**
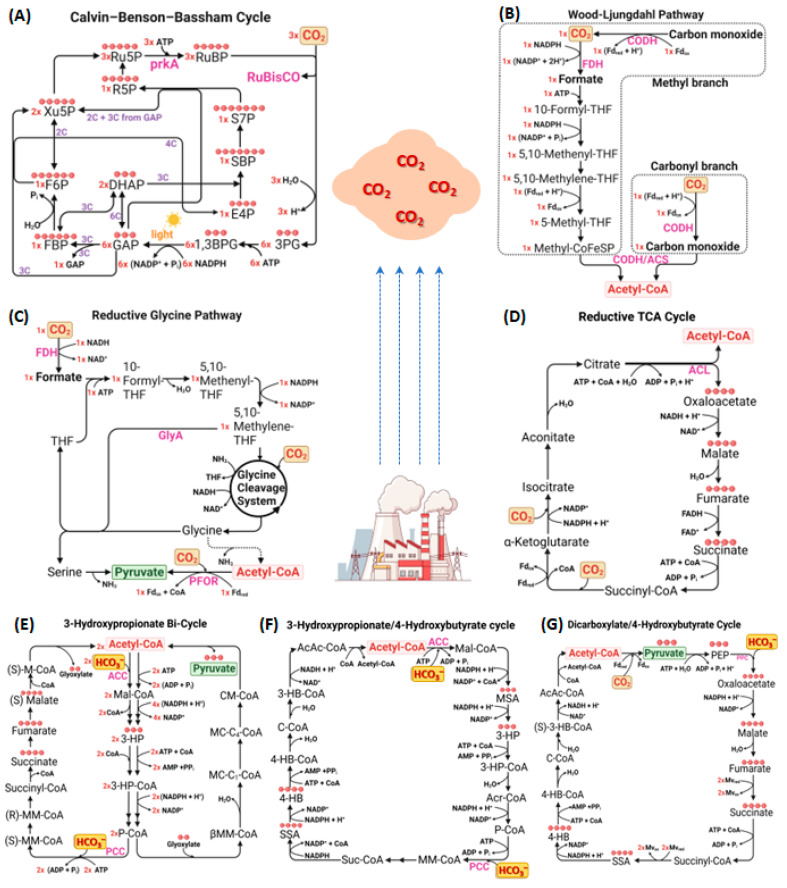
An overview of natural direct CO_2_ fixation pathways. (**A**) Calvin-Benson-Bassham (CBB) cycle; (**B**) Wood–Ljungdahl Pathway (WLP); (**C**) Reductive glycine pathway; (**D**) Reductive TCA cycle; (**E**) 3-Hydroxypropionate (3HP) Bicycle; (**F**) 3-Hydroxypropionate/4-Hydroxybutyrate (HP/HB) Cycle; (**G**) Dicarboxylate/4-Hydroxybutyrate (DC/HB) Cycle. Metabolites: ribulose 5-phosphate, Ru5P; ribulose 1,5-bisphosphate, RuBP; 3-phosphoglycerate, 3PG; 1,3-bisphosphoglycerate, 1,3BPG; glyceraldehyde 3-phosphate, GAP; fructose 1,6-bisphosphate, FBP; fructose 6-phosphate, F6P; xylulose 5-phosphate, Xu5P; dihydroxyacetone phosphate, DHAP; erythrose 4-phosphate, E4P; sedoheptulose 1,7-bisphosphate, SBP; sedoheptulose 7-phosphate, S7P; ribose 5-phosphate, R5P; tetrahydrofolate, THF; (3S)-citramalyl-CoA, CM-CoA; mesaconyl-C_4_-CoA, MC-C_4_-CoA; mesaconyl-C_1_-CoA, MC-C_1_-CoA; beta-methylmalyl-CoA, βMM-CoA; propionyl-CoA, P-CoA; 3-hydroxypropionyl-CoA, 3-HP-CoA; 3-hydroxypropionate, 3-HP; malonyl-CoA, Mal-CoA; (S)-malyl-CoA, M-CoA; (S)-methylmalonyl-CoA, S-MM-CoA; (R)-methylmalonyl-CoA, R-MM-CoA; acetoacetyl-CoA, AcAc-CoA; acryloyl-CoA, Acr-CoA; crotonyl-CoA, C-CoA; 4-hydroxybutyrate, 4-HB; 4-hydroxybutyryl-CoA, 4-HB-CoA; succinate semialdehyde, SSA; (S)-3-hydroxybutyryl-CoA, (S) 3-HB-CoA; malonate semialdehyde, MSA; phosphoenolpyruvate, PEP. Enzymes: Ribulose-1,5-bisphosphate carboxylase, RuBisCo; phosphoribulokinase, prkA; carbon monoxide dehydrogenase, CODH; acetyl-CoA synthase, ACS; formate dehydrogenase, FDH; serine hydroxymethyltransferase, GlyA; pyruvate synthase, PFOR; ATP-citrate lyase, ACL; acetyl-CoA carboxyltransferase, ACC; propionyl-CoA carboxylase, PCC; phosphoenolpyruvate carboxylase, PPC. Multi-step reactions are presented by continuous arrows. Special parts of WLP are shown dashed arrows. The figure was created with BioRender.

Several pathways facilitate the assimilation of atmospheric CO_2_ into organic materials, as shown in Figure 2. Among all natural CO_2_ fixation pathways, the Calvin–Benson–Bassham (CBB) cycle dominates and is responsible for 90% of global CO_2_ uptake [26]. Additionally, pathways such as the Wood–Ljungdahl (WLP), reductive glycine pathway (rGlyP), reductive tricarboxylic acid (rTCA) cycle, 3-hydroxypropionate bicycle (HP), 3-hydroxypropionate/4-hydroxybutyrate (HP/HB) cycle, and dicarboxylate/4-hydroxybutyrate (DC/HB) cycle play significant roles in CO_2_ utilization [27]. These processes, predominantly in autotrophic microorganisms, often lead to vital metabolites like pyruvate or acetyl-CoA, each with unique energy efficiency concerning ATP consumption [28].

##### Common Natural CO_2_ Fixation Cycles

**Calvin–Benson–Bassham (CBB) Cycle**: The CBB cycle stands as the premier identified biological CO_2_ fixation route and remains the primary carbon-fixation method in nature. Since it shares numerous metabolites and enzymes with the pentose phosphate pathway (PP pathway), it is also called the reductive PP pathway. Found in a variety of organisms such as plants, algae, cyanobacteria, and specific chemoautotrophic microorganisms, this cycle fundamentally operates through the enzymatic action of ribulose-1,5-bisphosphate carboxylase/oxygenase (RuBisCO). While RuBisCO’s central role in the CBB cycle is undeniable, its efficiency is often questioned. This enzyme catalyzes the transformation of ribulose 1,5-bisphosphate (RuBP) into 3-phosphoglycerate (3-PGA), but its efficiency is occasionally halved due to its tendency to favor O_2_ during photorespiration [29]. Known for its limited catalytic activity, RuBisCO’s O_2_ preference over CO_2_ is complicating endeavors aimed at engineering it for enhanced kinetics largely due to the intricate nature of its substrate-binding pocket [30]. However, the major efforts to enhance the cycle’s efficiency have been focused on engineering RuBisCO. For instance, a heterologous cyanobacterial RuBisCO was successfully overexpressed in *Ralstonia eutropha* (*Cupriavidus necator*), bolstering autotrophic growth and CO_2_ fixation capabilities [31]. Furthermore, a comprehensive in vitro examination of 143 RuBisCO enzyme activities unveiled a promising type-II RuBisCO variant from *Gallionella sp.*, which is iron-oxidizing chemolithotrophic bacteria [32]. In another recent study, 10 copies of RuBisCO were introduced by a delta-integration strategy into xylose-utilizing *Saccharomyces cerevisiae* and resulted in a 17% increase in ethanol yield and a 7% decrease in CO_2_ emission [33]. Such advancements underscore the potential to amplify CO_2_ assimilation rates by harnessing superior RuBisCO variants.

**Wood–Ljungdahl Pathway (WLP)**: The WLP, referred to as the reductive acetyl-CoA (rAc-CoA) pathway, is an exemplar of efficient non-photosynthetic carbon fixation. Requiring only one ATP molecule to produce pyruvate is notably more energy-conserving than the CBB cycle, which expends seven ATPs for the same result [5]. The WLP, primarily recognized in acetogens, operates exclusively under anaerobic conditions [34]. Microbes utilizing the rAC-CoA pathway often produce acetate or methane as end products [35]. Recently, Jang et al. constructed a *Clostridium acetobutylicum* strain to install heterologous WLP carbonyl branch genes from *Clostridium difficile* and performed CO_2_ fixation and increased biobutanol production [36].

**Reductive Glycine Pathway (rGlyP)**: The initial CO_2_ assimilation steps in the WLP parallel the reductive glycine pathway (rGlyP), which was first proposed to be synthetic, and then found to be natural [37]. This is because the rGlyP instead employs a glycine cleavage/synthase system (GCS) that incorporates CO_2_ and ammonium into 5,10-methylene-THF to produce glycine and recycle THF [28]. Highlighting their potential in microbial CO_2_ utilization, the WLP and the rGlyP stand out for their ATP efficiency in carbon fixation [38]. The most important advantage of the rGly pathway over the WLP is that the rGlyP can be operate both in aerobic and anaerobic microorganisms [39]. Strategies such as overexpressing the essential enzymes can further augment CO_2_ assimilation efficiency. For instance, *Eubacterium limosum*, when introduced with the GCS, exhibited an improved growth rate and acetate production [40]. Taking it further, even industrial microbes like *Pseudomonas putida* were engineered to assimilate CO_2_ and other C_1_-chemicals such as formate and methanol by introducing heterologous expression of the core-modules of the rGlyP [41]. With the help of adaptive laboratory evolution, a rGlyP-introduced formatotrophic *E. coli* strain was further developed to utilize CO_2_ and formate as sole carbon sources [42].

**Reductive Tricarboxylic Acid Cycle (rTCA)**: Initially discovered in the green sulfur bacterium *Chlorobium limicola*, the rTCA functions as the reverse counterpart to the traditional TCA (or Krebs cycle), primarily in strictly anaerobic or microaerobic autotrophic eubacteria [43]. Although studies on the rTCA cycle’s application in metabolic engineering remain limited, emerging research, such as one involving *E. coli*, has shown promising results in recycling CO_2_ and optimizing the production of acetate and ethanol [44].

##### Less Common Natural CO_2_ Fixation Cycles

**3-Hydroxypropionate (3HP) Bicycle**: The 3HP bicycle, or Fuchs–Holo bicycle, was first discovered in the thermophilic phototrophic bacterium *Chloroflexus aurantiacus* [45]. This cycle is considered unique due to its two cyclic CO_2_ assimilation pathways that collaboratively share initial reactions for CO_2_ assimilation, forming a complex bicyclic system. The 3HP bicycle consumes approximately 2.3 mol ATP to reduce 1 mole of CO_2_ to pyruvate, similar to the CBB cycle [46]. The 3HP bicycle’s key enzymes, such as propionyl-CoA synthase and malonyl-CoA reductase, have been leveraged to construct efficient cell factories for 3-hydroxypropionic acid [47]. Recently, the details of this uncommon mechanism have been revealed in filamentous anoxygenic phototrophs. Mesaconyl-CoA C_1_-C_4_ CoA transferase is found to catalyze the intramolecular CoA-transfer, which can be used for enzyme engineering to produce value-added chemicals [48].

**3-Hydroxypropionate/4-Hydroxybutyrate (HP/HB) Cycle and Dicarboxylate/4-Hydroxybutyrate (DC/HB) Cycle**: Remarkably, the HP/HB and DC/HB cycles, prevalent in certain archaea, demonstrate higher energy efficiency in anaerobic environments, with the DC/HB cycle being particularly efficient, requiring only 1.6 mol ATP to reduce one mol CO_2_ to pyruvate [46]. From an evolutionary perspective, the capability of the 3HP bicycle and the HP/HB cycle to assimilate bicarbonate rather than CO_2_ is notable. This adaptability likely stems from the higher intracellular concentration of bicarbonate compared to CO_2_. This feature and oxygen tolerance potentially contribute to their evolutionary survival [49]. From an application standpoint, there have been attempts to harness these pathways for biotechnological purposes. (S)-3-hydroxybutyryl-CoA dehydrogenase, which is one of the important enzymes of the HP/HB cycle, has been characterized, and different enzymes from *Nitrosopumilus maritimus* and *Metallosphaera sedula* were compared to explore the enzymatic differences in these processes within the DC/HB and HP/HB cycles, which helps protect marine habitats [50]. However, attempts to fully recreate and utilize these pathways in common microbial hosts like *E. coli* have faced challenges [5].

#### 2.1.2. Synthetic CO_2_ Fixation Pathways

Synthetic CO_2_ fixation pathways have garnered significant attention as potential alternatives to enhance carbon assimilation efficiency, transcending the inherent constraints observed in natural pathways. The focus lies in developing pathways with optimized thermodynamic and kinetic properties while overcoming difficulties associated with key enzymes like RuBisCO [30,32]. One noteworthy example is the crotonyl-CoA/ethylmalonyl-CoA/hydroxybutyryl-CoA (CETCH) cycle. Assembled using 17 enzymes derived from nine distinct organisms, the CETCH cycle has displayed a greater rate of CO_2_ fixation and a reduced ATP requirement compared to the CBB cycle [26]. Its efficiency is partly attributed to the use of the enoyl-CoA carboxylase/reductase enzyme, which showcases high carboxylation activity. However, translating the in vitro success of the CETCH cycle into in vivo applications remains a challenge [49]. To overcome this challenge, the same group developed a new pathway called the HydrOxyPropionyl-CoA/Acrylyl-CoA (HOPAC) cycle, which consists of 11 enzymes from 6 different organisms and is similar to the natural 3HP cycle but with the introduction of new in-between reactions to increase the ATP efficiency to 33% [51].

Another synthetic CO_2_ assimilation route is the Gnd–Entner–Doudoroff (GED) pathway. By inducing specific gene deletions in *E. coli*, researchers demonstrated the energy-efficient reductive carboxylation of ribulose-5-phosphate via this pathway. Despite its potential, the complete cyclic GED pathway has only been partially shown in vivo [52]. Another advancement was made when researchers synthesized starch from CO_2_ and hydrogen in a cell-free system. This process coined the artificial starch anabolic pathway (ASAP), comprised 11 core reactions, and showcased an impressive CO_2_-to-starch conversion rate. This rate was approximately 8.5 times faster than starch synthesis observed in corn [53]. Since pathway length also generates problems for energy efficiency, novel pathways like the POAP cycle and the ICE-CAP pathway have been proposed [54]. The POAP cycle, comprising merely four steps, potentially offers a more streamlined and efficient approach to carbon sequestration. The ICE-CAP pathway, on the other hand, utilizes CO_2_ alongside high-energy C1 compounds, such as methanol or formaldehyde, obviating the need for ATP and cofactors like NAD(P)H [55].

One computational study, utilizing a repository of around 5000 known enzymes, unveiled the Malonyl-CoA-Oxaloacetate-Glyoxylate (MOG) pathways. These proposed pathways, which display ATP efficiency over the conventional CBB, might be revolutionary. They use rapid carboxylases and are oxygen-tolerant. However, some enzymes in MOG pathways are thermally sensitive, and their end-product, glyoxylate, when integrated into central metabolism, could revert to CO_2_, causing this study performed only in in silico [56]. Nevertheless, designing and implementing synthetic pathways is not without its challenges. When introduced into diverse microbes, these synthetic pathways can disrupt the metabolic balance, necessitating further optimization to realign central metabolic fluxes. Despite this, the capabilities of these synthetic pathways, especially when combined with other technological advancements like biocompatible semiconductor materials or cell-free systems, offer promising avenues for the future of carbon sequestration and utilization [57].

#### 2.1.3. Host Selection and Reducing Power

##### CO_2_-Fixing Autotrophs and Synthetic Hosts

Microorganisms that can synthesize organic substances by fixing inorganic carbon, leveraging energy from either light or inorganic chemicals, are classified as autotrophs. Depending on their energy source, these autotrophs bifurcate into two groups: photoautotrophs, which harness energy via photosynthesis, and chemoautotrophs, which extract energy from chemical reactions [58].

Photoautotrophs, such as cyanobacteria and microalgae, derive energy from photosynthesis. These organisms house photosynthetic pigments, allowing them to harness energy from light and water [59]. Notably, they assimilate CO_2_ primarily via the Calvin–Benson–Bassham (CBB) cycle. Due to their superior solar energy utilization and rapid growth rates compared to terrestrial plants, they have gained considerable attention as potential bioproduction platforms [60]. Cyanobacterial strains like *Synechocystis* sp. and *Synechococcus* sp., for instance, have made significant strides in metabolic engineering, and these advancements enable them to produce valuable chemicals [61,62]. Furthermore, certain eukaryotic microalgae have been explored for lipid and alkane production, though their genetic manipulation is somewhat restricted due to limited transformation efficiencies and genetic tool availability [63].

On the other hand, chemoautotrophs, including certain bacteria, obtain energy through chemical reactions. A prominent example is the hydrogen-oxidizing bacteria *Cupriavidus necator*, which can oxidize substances like H_2_ [64] or formate [65]. This bacterium is known for its ability to naturally accumulate polyhydroxybutyrate (PHB), a precursor for bioplastics, comprising up to 70% of its biomass [66]. Furthermore, genetic engineering has expanded its repertoire to produce chemicals such as branched-chain alcohols and alkanes [67,68]. Another chemoautotroph of interest is *Acidithiobacillus ferrooxidans*, which can absorb electrons from Fe^2+^ or directly from a cathode in bioelectrochemical systems [69].

Acetogens represent another subset of chemoautotrophs, which are strictly anaerobic bacteria and use specifically WLP. Certain acetogens, like *Clostridium ljungdahlii*, *Clostridium autoethanogenum*, and *Acetobacterium woodii*, are naturally equipped to produce chemicals such as acetate, ethanol, and 2,3-butanediol [70]. Genetic tools have been applied to acetogens to expand their production portfolio, with some species even being utilized for large-scale industrial applications [71]. Yet, their ATP regeneration capacity poses challenges in producing ATP-intensive products.

In heterotrophic hosts, organisms like *E. coli* and *S. cerevisiae* do not initially possess functional CO_2_ fixation pathways or photosystems. However, scientific endeavors have partially succeeded in transplanting such systems into these hosts, thus ushering in a mixotrophic mode of nutrition [72]. Shifting the spotlight to synthetic autotrophic microorganisms, model organisms like *E. coli*, *Saccharomyces cerevisiae*, and *Corynebacterium glutamicum* have been engineered to metabolize CO_2_. For instance, *E. coli* has been engineered to fix CO_2_ by co-expressing RuBisCO, phosphoribulokinase, and FDH, using formate as a reducing agent [73]. On the other hand, *S. cerevisiae*, despite the successful expression of RuBisCO from *Cupriavidus necator*, has failed to grow on sole CO_2_ [74]. Recent advances have also demonstrated that autotrophic production platforms can effectively integrate autotrophic and heterotrophic hosts, melding their beneficial traits. A notable instance involves the non-engineered autotrophic acetogen *Sporomusa ovata* paired with engineered *E. coli* strains. *S. ovata*, harnessing semiconductor nanowires, fixes CO_2_ and excretes acetate—a substrate-engineered *E. coli* strain that can produce valuable compounds like n-butanol or PHB under aerobic conditions; up to 52% of acetate-to-product yield was reported for PHB production by *E. coli* [75]. Similarly, another two-reactor system combines the thermophilic acetogen *Moorella thermoacetica* and yeast *Yarrowia lipolytica*, where the former’s acetate output serves as a feedstock for the latter, engineered for increased lipid synthesis [76]. Such systems still need improvement converting CO_2_ into valuable end products, achieving sustainable energy conversion efficiencies.

Successfully applying microbial hosts with CO_2_ fixation capabilities depends on deeply understanding their physiology, biochemistry, and genetics. Both photoautotrophic and chemoautotrophic microbes offer unique opportunities for bioproduction, with advances in genetic tools and metabolic engineering paving the way for more efficient autotrophic cell factories. These microbial systems, in combination with advances in metabolic engineering, hold immense potential to revolutionize the sustainable production of value-added compounds.

##### Energy Supplies for Microbial CO_2_ Fixation

Reducing powers such as NAD(P)H, FADH, ferredoxin red (Fd_RED_), and menaquinol serve as driving forces in microbial CO_2_ fixation, which is pivotal for metabolism. Regeneration of these reducing powers entails the extraction of high-energy electrons from either organic and/or inorganic compounds, or light. Light remains the most prevalent energy source utilized by photoautotrophs like plants, algae, and photosynthetic microorganisms [77]. Photosystems I and II (PS I and PS II) are the primary photo-reaction complexes in photolithotrophic organisms like plants, algae, and cyanobacteria [78]. They absorb light wavelengths ranging from 400 to 700 nm, facilitating the photocatalytic splitting of water to produce ATP and NADPH, thereby providing the requisite energy for CO_2_ fixation [79]. PS I absorbs light and uses it to excite a low-energy electron from chlorophyll, which then produces Fd_RED_ and eventually NADPH. PS II compensates for the electron extracted from PS I by a subsequent electron transfer, originally sourced from a water-splitting reaction [78]. Recently, *Chroococcidiopsis thermalis* has demonstrated growth in far-red light through specialized photosystems, highlighting the potential for engineering increased efficiency in light utilization [80]. However, there is an inherent energy loss of around 60% in the electron transfer between PS I and II, limiting the efficiency of this system [81]. Efforts to address this inefficiency include the integration of artificial photosensitizers, such as incorporation of cadmium sulfide nanoparticles with *Moorella thermoacetica* to facilitate the photosynthesis of acetic acid from CO_2_ [82].

On the other hand, chemolithotrophs utilize inorganic compounds to extract high-energy electrons for regenerating their reducing powers. The hydrogen-oxidizing bacteria, for instance, employ hydrogenases to consume H_2_ and regenerate reducing powers. These hydrogenases come in two known varieties: membrane-bound, which uptake hydrogen to produce ATP, and soluble NAD-reducing hydrogenases, which produce NADH [83,84]. For example, *E. coli* possesses membrane-bound hydrogenases, with Hyd-1 or Hyd-2 catalyzing hydrogen uptake to generate ATP [85]. *Ralstonia eutropha*, a natural hydrogen-utilizing autotroph, has been studied for its hydrogenase-driven ATP and NADH generation, which, expressed as in the soluble hydrogenase form in *E. coli*, have shown promise in enhancing intracellular NADH levels [86]. As another example for inorganic compounds to exploit high-energy electrons, iron-oxidizing bacteria oxidize Fe^2+^ ions to generate NADH [87]. Meanwhile, nitrifying bacteria like ammonia-oxidizing bacteria and nitrite-oxidizing bacteria obtain high-energy electrons by oxidizing nitrogen compounds [88,89]. Notably, sulfur-oxidizing bacteria, derive their electrons from the oxidation of various sulfur compounds through intricate pathways to regenerate reducing powers such as menaquinol, NADH, and Fd_RED_ [90]. A smaller group of bacteria focuses on the oxidation of PO_3_^3−^ to PO_4_^3−^, using phosphite dehydrogenase to transfer electrons and regenerate NADH [91].

In summary, microbial CO_2_ fixation relies heavily on various pathways to regenerate essential reducing powers, utilizing light and chemicals as energy sources. Whether through photosystems in photoautotrophs or hydrogenases in chemolithotrophs, these microorganisms have developed diverse mechanisms to ensure efficient CO_2_ fixation, underpinning their importance in the planet’s carbon cycle. To regenerate more reducing power, using renewable electricity can also be one of the options for both keeping the carbon-neutral environment and regenerating more reducing agents, as mentioned in detail in the next subsection.

#### 2.1.4. Microbial Electrosynthesis 

**Figure 3:**
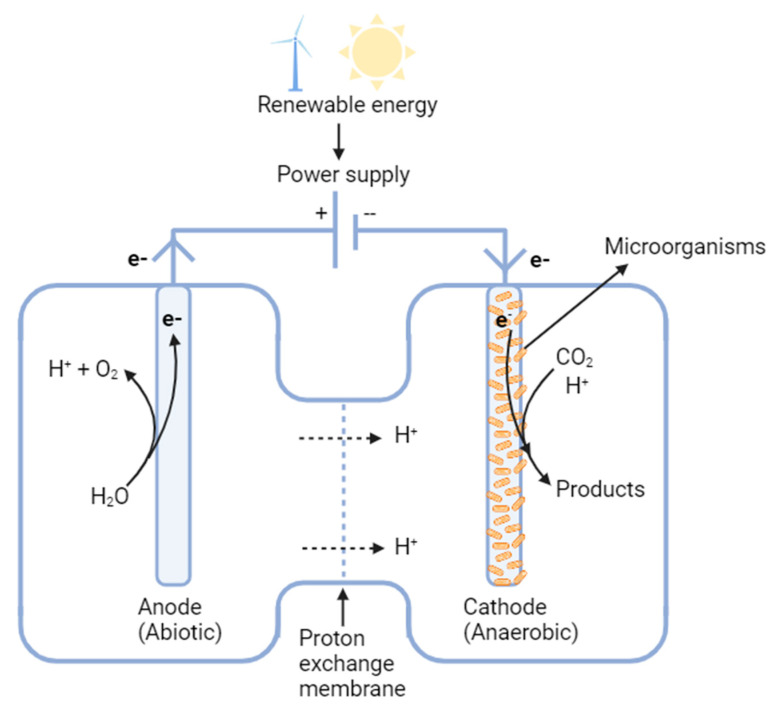
A brief summary of the mechanism of microbial electrosynthesis that can be used for one-step CO_2_ fixation and conversion (remade from the reference of [95]). The figure was created with BioRender.

As shown in Figure 3, microbial electrosynthesis (MES) is an innovative bioelectrochemical approach that leverages electroactive microorganisms to convert renewable electrical energy into value-added products [92,93]. Rooted in bioelectrochemical systems (BES) principles, MES offers a sustainable route to harness CO_2_ for the synthesis of biofuels and commodity chemicals, some of which include methane, acetate, formic acid, and ethanol, among others, potentially mitigating the detrimental impacts of CO_2_ emissions [94]. At its core, MES operates by utilizing a biofilm on an electrode as a catalyst, which contrasts with traditional methods that employ chemical catalysts [23].

The MES architecture is intricate [96]. The anodic chamber operates abiotically, where water undergoes splitting to generate protons, electrons, and oxygen. Electrons generated in this chamber are channeled toward the biocathode via an external circuit when an external voltage is applied to the electrochemical cell. Conversely, electrophilic bacteria, primarily acetogens, inhabit the cathodic chamber, which maintains anaerobic, biotic conditions. CO_2_ acts as an electron acceptor in the MES system, undergoing fixation and conversion at the cathode [97]. Certain electroactive microbes have demonstrated the ability to shuttle electrons intra- and extra-cellularly in this environment [98]. Herein, specialized microbes like *Sporomusa* species and engineered strains of *Clostridium* have exhibited the potential to generate biofuels directly from CO_2_ [99,100]. A classic example demonstrates an acetate production rate of 142.2 mg/L/d and a carbon conversion efficiency of 84% when utilizing enriched mixed homoacetogenic bacteria [101]. Notably, other microbes such as *Clostridium scatologenes* ATCC 25,775 employ the WLP pathway for CO_2_ fixation, generating acetic acid, butyric acid, and ethanol by using H_2_ as reducing power [102].

The true potential of MES lies in its scalability and flexibility. The efficiency and spectrum of products from MES can be influenced by adaptive measures like improved electrode materials, specialized bioreactor designs, and genetically engineered biocatalysts [103]. Indeed, bioreactor optimization, which included strategies like increasing biomass retention and media dilution rate, showcased an acetate production with a titer of 13.5 g/L [104]. Beyond acetate, MES also promises the generation of other valuable bioproducts like butyrate, caproate, and polyhydroxybutyrate (PHB) [105,106,107]. 

However, MES also faces challenges for wider applications. Current systems grapple with issues like low CO_2_ conversion rates, high-energy input, and the nuances of maintaining effective microbial communities [108]. Fortunately, recent innovations have exhibited promise to enhance system efficiency. For instance, thermal conditions have been found to influence these processes; *Moorella thermoautotrophica* exhibited an enhanced rate of acetate and formate production at 55 °C as opposed to 25 °C [109]. The microbes’ biodiversity in MES also plays a pivotal role in its efficiency. Notably, autotrophic sulfate-reducing bacteria (SRM) have displayed potential as excellent biocatalysts, elevating the performance of BES in CO_2_ fixation [110]. These bacteria hold the potential to improve hydrogen production and water sulfate removal. In a recent study, a co-culture of *Desulfopila corrodens* and *Methanococcus maripaludisco* magnified methane production twenty-fold compared to *M. maripaludisco* alone [111]. Electro-catalyst-assisted MES systems have been developed with electrical-biological hybrid cathodes to improve product rates and variety. Here, Zn-based electrodes have outperformed others; one system achieved an acetic acid production rate of 1.23 g/L [112].

Overall, the CO_2_ bioelectrorefinery concept, as heralded by MES, is an embodiment of a circular bioeconomy, envisioning an integration of CO_2_ capture, renewable energy, and sustainable production of chemicals and fuels [113]. While strides have been made, the commercial realization of MES awaits advancements in electrode materials, microbial communities, and process optimization to rival traditional biomass-based processes. Nevertheless, the trajectory of MES research promises a sustainable and innovative path to a cleaner, greener future [114].

### 2.2. Two-Step Strategy—Fixing CO_2_ into C_1_/C_2_ Chemicals via Electrochemical Catalysis and Converting C_1_/C_2_ Chemicals into Bioproducts via Biomanufacturing 

The two-step/indirect CO_2_ fixation and conversion strategy takes the advantages of the current advances from both electrochemical CO_2_ fixation into C_1_/C_2_ chemicals and the synthetic biology to further convert the derived C_1_/C_2_ chemicals into the fuels, chemicals, and pharmaceuticals via biomanufacturing process. A primary advantage of these substrates is their non-competitive nature with alimentary resources, which contributes to an economically sustainable framework while diminishing carbon efflux into the biosphere [115]. Nevertheless, it has been widely studied that the C_1_/C_2_ substrates can be produced from CO_2_ via an electrochemical catalysis process [116], which uses renewable electricity from solar, wind, or hydraulic power to capture and fix CO_2_ into specific C_1_/C_2_ products at high yield and selectivity. This two-step CO_2_ fixation and conversion approach can potentially reduce the dependence on fossil oil-based fuels and chemicals and mitigate the impact of greenhouse gas emissions on the environment [117]. 

#### 2.2.1. Using CO_2_-Derived C_1_ Chemicals for Biomanufacturing

Gaseous one-carbon (C_1_) substrates like CO and CH_4_ are from industrial wastes like steel mills and biomass gasification, whereas liquid C_1_ substrates, such as formate and methanol, are derived from CO_2_ or waste gas conversions [118]. As the direct CO_2_ splitting into CO and oxygen is a thermodynamically unfavorable reaction due to the stability of CO_2_ at ambient temperatures, the response demands a large amount of energy for initiation [119]. Although this reaction was attempted to be feasible by membrane reactor systems by lowering the energy input, the conversion rates are too low to be efficient at an industrial scale. Moreover, conversion efficiencies might cause futile separation of the resultant products, CO and O_2_, to handle at higher temperatures [120]. Initiatives have been undertaken to capture CO_2_ and transform it catalytically into a range of high-value products by employing hydrogenation and oxidation processes. However, these chemical conversions of C_1_ compounds pose significant challenges, including costly catalysts, extreme conditions such as high temperatures (around 450 °C) and pressures (approximately 30 MPa), and the emission of hazardous by-products such as carbon monoxide. These factors contribute to the overall expense and unsustainability of the technology [121]. 

**Figure 4:**
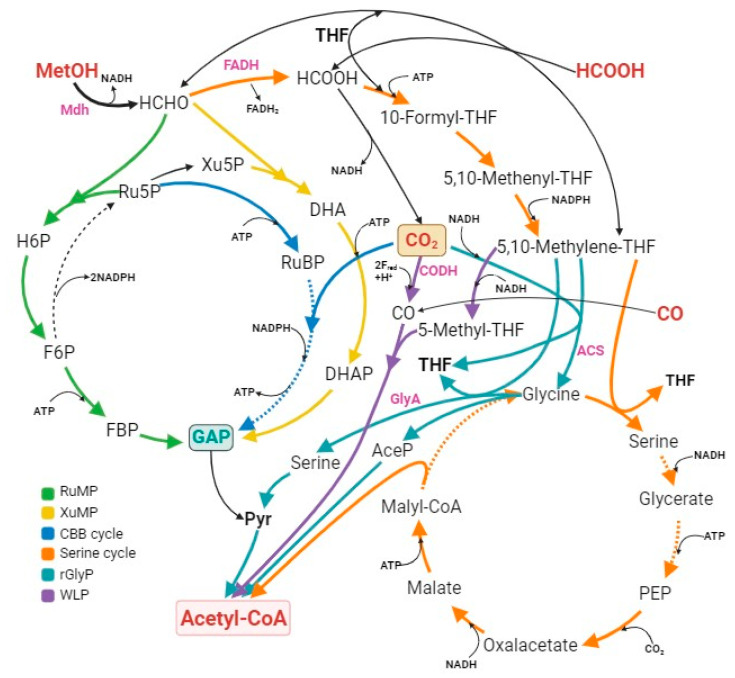
Typical C_1_ utilization pathways. Metabolites: ribulose 5-phosphate, Ru5P; hexulose 6-phosphate, H6P; glyceraldehyde 3-phosphate, GAP; fructose 6-phosphate, F6P; fructose 1,6-bisphosphate, FBP; xylulose 5-phosphate, Xu5P; dihydroxyacetone, DHA; ribulose-1,5-bisphosphate, RuBP; tetrahydrofolate, THF; 3-phosphoglycerate, 3PG; 1,3-diphosphoglycerate, 1,3DPG; phosphoenolpyruvate, PEP; pyruvate, Pyr. Enzymes: carbon monoxide dehydrogenase, CODH; acetyl-CoA synthase, ACS; membrane-bound methane monooxygenase, pMMO; cytoplasmic methane monooxygenase, sMMO; alcohol oxidase, Aox; methanol dehydrogenase, Mdh; formaldehyde dehydrogenase, FADH; formate dehydrogenase, FDH; serine hydroxymethyltransferase, GlyA; Ribulose-1,5-bisphosphate carboxylase, RuBisCo. Multi-step reactions are presented with dashed arrows. Special parts of WLP are shown in faded dashed arrows in the related color. The figure was created with BioRender.

The liquid C_1_ substrates are advantageous as they are storable and fully soluble, supporting higher production. Microbes can transform C_1_ substrates into products like alcohols, acids, and plastic components. Specific bacteria can process CO or CH_4_ and create multi-carbon compounds [122]. Some also use formate and methanol, which are essential in the C_1_ pathway [118]. In the following section, natural autotrophs and industrial strains that have been engineered to fix CO_2_ and recent advances in molecular biology and metabolic engineering for creating more effective CO_2_ fixation pathways will be discussed. Typical C_1_ chemical fixation pathways are shown in Figure 4.

##### Carbon Monoxide 

Carbon monoxide (CO) is a relatively rare gas in the atmosphere, but novel electrochemical CO_2_ conversion approaches can effectively produce CO from CO_2_ [123]. Waste gases from industrial processes partially oxidizing carbon-containing compounds or gasifying waste streams can also yield CO [5]. The co-electrolysis of CO_2_ and H_2_O can also produce CO. One of the primary concerns of using CO is its high toxicity and difficulty in detection because it is colorless, odorless, and tasteless [124]. Although CO has the potential to impair oxygen transport and mitochondrial function in many organisms, it can be an advantageous carbon and energy source for a phylogenetically diverse array of bacteria and archaea known as carboxydotrophs [125]. Carboxydotrophs have evolved to assimilate CO using carbon monoxide dehydrogenase (CODH), which catalyzes CO oxidation to CO_2_, providing reducing power to the cell and employing either molybdenum (for aerobes) or nickel (for anaerobes) as essential metal cofactors to facilitate electron transport [126,127].

In aerobic carboxydotrophs, the generated CO_2_ is typically assimilated via the Calvin–Benson–Bassham (CBB) cycle to produce biomass. Aerobic CO oxidation, which is more exothermic and possesses higher free energy (ΔG0 = −514 kJ) than anaerobic CO oxidation (ΔG0 = −174 kJ), is advantageous for synthesizing ATP-intensive complex products, thereby facilitating higher ATP availability and resulting in increased biomass concentrations [128]. Recent studies have shown the potential of aerobic production of complex molecules, such as the production of C_15_ sesquiterpene (E)-α-bisabolene from synthesis gas (syngas), a composite of CO, H_2_, CO_2_, and trace amounts of impurities such as H_2_S and NH_3_—in *Hydrogenophaga pseudoflava*, although there are challenges due to the potentially explosive mixture of O_2_ and CO [129].

Anaerobic carboxydotrophs predominantly employ the WLP pathway, also known as the reductive acetyl-CoA pathway, for CO and CO_2_ assimilation [127]. The WLP bifurcates into two branches: the carbonyl branch, which reduces CO_2_ to CO, and the methyl branch, which transforms CO_2_ into formate and its subsequent products. This pathway has garnered significant attention in biotechnological research and genetic and metabolic engineering, particularly in relation to acetogens, microorganisms that use the WLP as their signature pathway [130]. Despite some progress, it remains challenging to demonstrate growth in CO and nonacetogenic hosts. Initial attempts failed to demonstrate CODH/acetyl-CoA synthase (ACS) activity in *E. coli* by expressing genes from *Morella thermoacetica* [131]. Success was later achieved following the incubation of ACS in NiCl_2_ solution, although growth using CO as a substrate remained elusive. One major obstacle is the inadequate intracellular conditions and genetic framework of traditional hosts, such as *E. coli* or yeast, which limits the production and assembly of essential cofactors and sensitive metal centers [123]. As a different strategy, hosts and gene sources with closer phylogenetic relationships have been employed. In recent study, a group of genes from *Clostridium ljungdahlii*, responsible for encoding CODH/ACS, in conjunction with a methylenetetrahydrofolate reductase gene from *E. coli*, were integrated into *C. acetobutylicum* [132]. This reconstruction enabled functional WLP, thereby underscoring the crucial role played by metal clusters. Another study demonstrated increased CO oxidation rates (3.1-fold) through overexpression of the endogenous CODH/ACS complex in *Eubacterium limosum* [133]. In addition, specific adaptive laboratory evolution attempts in CODH or ACS have been proven to enhance the activity of the CODH/ACS complex for CO oxidation, showing higher growth and CO gas uptake rates [134]. Nonetheless, despite these advancements, the complete transformation of non-acetogenic microorganisms into carboxydotrophs requires further research.

##### Methane 

Methane (CH_4_) is a potent greenhouse gas, ubiquitous in natural and shale gas reserves. Anthropogenic methane sourced from human activities, including landfills, agricultural practices such as animal livestock emissions, paddy rice cultivation, coal mining, and wastewater treatment, contributes significantly to global warming [135]. According to estimates from the Environmental Defense Fund, at least 25% of present-day global warming is attributable to anthropogenic CH_4_ emissions. This is a significant concern because CH_4_, over the initial two decades following its release into the atmosphere, exhibits a warming effect over 80 times greater than CO_2_ [136]. Consequently, cultivating CH_4_ for biotechnological applications has dual implications: it not only enhances its value beyond traditional uses, such as generating heat or electricity (termed revalorization), but also plays a pivotal role in curbing greenhouse gas emissions.

CH_4_ assimilation is initiated by converting methane monooxygenase (MMO) to methanol. Methanotrophs, organisms capable of metabolizing methane exclusively as their carbon source in oxygen-rich and oxygen-deprived environments, have two separate versions of MMO. One is a soluble intracellular variant (sMMO), and the other is a particulate form attached to the membrane (pMMO) [137]. Once methanol is produced, it undergoes further oxidation to form formaldehyde. This compound can then be broken down into CO_2_, which involves specific enzymes, notably formaldehyde dehydrogenase and formate dehydrogenase [138]. Some intermediate formate or formaldehyde is integrated via serine or ribulose monophosphate (RuMP) cycles, serving as a carbon source in the biomass. Formaldehyde is utilized in the RuMP cycle, transforming it into hexulose-6-phosphate and later into ribulose-5-phosphate to complete the cycle. Through the (tetrahydromethanopterin) H4MPT pathway, formaldehyde undergoes a conversion process to become formate. Meanwhile, the serine cycle incorporates formate through the (tetrahydrofolate) H4F pathway and finally converts serine into glycine to close the cycle [139].

sMMO is recognized for its extensive substrate specificity; however, high copper concentrations may adversely affect its performance. Conversely, pMMO, owing to its proximity to the membrane, has superior accessibility to methane compared to sMMO. The linkage of pMMO with the membrane indicates its ability to accelerate catalysis in CH_4_ oxidation mechanisms [140]. The phenomenon of anaerobic CH_4_ oxidation first came to light within microbial consortia. In these communities, the transition of methane to CO_2_ was paired with the reduction in specific elements, such as sulfate, nitrate, nitrite, iron, or manganese [141,142,143,144]. However, owing to difficulties in obtaining pure cultures, all methanotrophs identified to date are aerobic bacteria [145]. Methanotrophs have been metabolically engineered to yield value-added chemicals from CH_4_, such as lactate, succinate, and astaxanthin [146]. Despite the slower development and growth rates of methanotrophs, non-native hosts, such as *Escherichia coli*, offer promising potential for CH_4_ utilization owing to a deeper understanding of their physiology and established metabolic engineering systems [147].

Utilizing industrially relevant strains, such as *E. coli*, for methane bioconversion is a promising strategy because of its superior growth rate, in-depth understanding of its physiology, wide range of system/synthetic tools available, and well-established metabolic engineering system for value-added products. However, achieving the full activity expression of methane monooxygenases in non-native hosts has proven to be a largely unsuccessful challenge thus far [148]. Protein engineering endeavors have used P450 monooxygenase as an alternative to MMO for converting methane to methanol; however, these attempts have garnered very limited success [149,150]. The only progress made includes the expression of the β-subunit of pMMO in *E. coli*, albeit with merely detectable activity [151]. This underscores that the principal challenge in synthesizing methanotrophs in non-native hosts depends on the functional expression of the enzyme responsible for methane oxidation. Despite these obstacles, recent breakthroughs have led to promising outcomes. For example, the β-subunit of pMMO and the catalytic domains of pMMO from *Methylococcus capsulatus* have been effectively expressed as soluble enzymes in *E. coli*. By reassembling these enzymes in a framework built from apoferritin particles, a pMMO-mimetic enzyme particle was generated. This assembly exhibits in vitro methanol production kinetics that rival those of native pMMO [152]. Additionally, heterologous expression of sMMO from *M. capsulatus* and the GroESL chaperone CH_4_ was converted to acetone in an *E. coli* strain previously engineered for methanol-based acetone production [153]. These advances indicate the proof-of-concept and feasibility of synthetic microbes for CH_4_ bioconversion, suggesting that further strain engineering could significantly enhance the conversion rates and yields, potentially fulfilling the industrial potential of microbial CH_4_ bioconversion.

##### Methanol

As of 2018, the worldwide methanol (CH_3_OH) production capacity stood at around 100 million metric tons annually, demonstrating a steady increase in the capacity to convert CH_4_ into methanol and a concurrent decrease in methanol prices [154]. Today, methanol’s cost is already comparable to glucose, an outcome largely influenced by its production predominantly from natural gas, crude oil, and coal via methods such as steam reforming of natural gas, biomass-derived synthesis gas, or through hydrogenation of CO_2_; this makes its price ($150–300/ton) generally lower than that of sugar ($300–400/ton) [9,10,155]. Methanol, significantly more reduced than most sugars, is an attractive substrate or co-substrate alongside sugars for producing various metabolites, including alcohols, carboxylic acids, fatty acids, and hydrocarbons, given its high reductivity. It boasts a reduction degree of six per carbon, compared to glucose’s four, denoting that methanol possesses 50% more electrons per carbon atom, thus housing a high-energy content. This abundance of electrons can be harnessed to boost product yields in fermentations, further accentuating methanol’s appeal as a substrate [156].

Among all identified native methylotrophy groups, aerobic methylotrophy is the largest, encompassing both prokaryotic and eukaryotic forms, represented by well-studied bacteria such as *Bacillus methanolicus* and the *Methylobacterium extorquens*, as well as certain yeast species like *Pichia pastoris* [157]. These aerobic methylotrophs employ two key methanol-utilization pathways. The initial pathway involves the oxidation of methanol to formaldehyde, facilitated by three classes of oxidoreductases, each distinguished by their electron acceptors: PQQ-dependent methanol dehydrogenases (MDHs), NAD^+^-dependent MDHs, O_2_-dependent alcohol oxidases (AODs) [158]. The first two are primarily found in methylotrophic bacteria, while the latter is characteristic of methylotrophic yeasts [159]. NAD^+^-dependent MDHs stand out for their ability to use a universal cofactor, NAD^+^, to transfer electrons for metabolite production, creating reducing equivalents of NADH. O_2_-dependent AODs, identified mainly in yeasts, convert methanol into hydrogen peroxide and formaldehyde [160]. The second pathway entails the incorporation of formaldehyde into central carbon metabolism via one of three identified assimilation pathways in aerobic methylotrophs: the xylulose monophosphate (XuMP) cycle (as known as dihydroxyacetone (DHA) cycle), the RuMP cycle, and the serine cycle. The XuMP pathway predominantly occurs in yeasts, while the RuMP and serine pathways are observed in *B. methanolicus* and *M. extorquens*, respectively [161,162]. The serine pathway stands out for its carbon efficiency, fixing 3 mol CO_2_ and merging 3 mol formaldehyde to produce 3 mol acetyl-CoA, although it is also the most ATP-costly. In contrast, the RuMP pathway exhibits the highest energy efficiency, generating 2 mol of NADH and 1 mol of ATP per mole of acetyl-CoA. The XuMP pathway, meanwhile, yields 2 mol of NADH but consumes 1 mol of ATP per mole of acetyl-CoA produced [163].

Anaerobic methylotrophy is mainly limited to methanogenic archaea and acetogenic bacteria, with the latter gaining interest due to their metabolic capacity for high acetate or butyrate production [164]. In methylotrophic acetogens, the methyl-THF produced by the methyltransferase system enters the WLP pathway to generate cell mass and conserve energy [165]. The WLP consists of two separate branches, the methyl, and the carbonyl, each handling one CO_2_ molecule. In the methyl branch, CO_2_ converts to formate, which merges with auxiliary tetrahydrofolate and reduces to the methyl group of tetrahydrofolates, a precursor for the methyl group of acetyl-CoA. Conversely, in the carbonyl branch, CO_2_ transforms to CO, merging with methyl-THF from methanol to produce acetyl-CoA via the CO dehydrogenase/acetyl-CoA synthase (CODH/ACS) [166]. This resultant acetyl-CoA can be used for pyruvate synthesis, biomass production, or acetate generation, enabling ATP production [58]. With higher energy efficiency in converting methanol to biomass or products than aerobic methylotrophs, anaerobic acetogens can also assimilate other C_1_-compounds such as CO_2_ and CO due to the presence of the WLP pathway. This methanol assimilation also involves CO_2_ fixation, making acetogens attractive platform microbes for methanol bioconversion [122]. 

Native methylotrophs hold the potential for generating high-value chemicals from methanol, but methanol assimilation rates curb the efficiency [167]. Expanding these rates to produce target compounds is an insistent need. While the limited availability of genetic tools poses a challenge, the strides made in synthetic biology now enable the development of these tools to engineer native methylotrophs [168]. For example, *B. methanolicus* was modified to generate L-lysine by implementing the CRISPRi system [169]. Similarly, *M. extorquens* was enabled to produce itaconate by heterologously introducing the cis-aconitic acid decarboxylase gene from *Aspergillus terreus* [170]. For the aerobic methylotrophs, intermediate metabolite formaldehyde accumulation may cause cellular toxicity due to the macromolecule interactions [18]. Anaerobic acetogens are favorable to avoid formaldehyde toxicity since methanol is directly assimilated through WLP [158]. Similarly, methanol assimilation is conducted within the peroxisome in methylotrophic yeasts, and this might have an advantage over other microbes in keeping the formaldehyde away from other cell components [9]. For example, *P. pastoris* could produce free fatty acids with superior efficiency from methanol by boosting the availability of precursors and cofactors and minimizing the buildup of formaldehyde through optimized methanol metabolism engineering [171]. Another known methylotrophic yeast, *Ogataea polymorpha* growth, was also restrained by formaldehyde formation. Engineering pentose phosphate (PP) and gluconeogenesis pathways and further ALE efforts overcome those problems and implemented efficient free fatty acid production with a titer of 15.9 g/L [172]. Nonetheless, more efforts in genetically engineering the native methylotrophs are required due to constraints like the insufficient understanding of cellular metabolic pathways and a confined set of genetic tools for such engineering [165].

Initiatives have been directed toward creating synthetic methylotrophs to navigate the abovementioned challenges. For instance, by integrating the heterologous methanol assimilation pathway from *B. methanolicus* MGA3 into *Bacillus subtilis*, a methanol-dependent engineered strain that can process 4.09 g/L methanol was produced [173]. In addition to integrating natural methanol-utilization pathways into non-native hosts, unique enzymatic conversions have been employed in synthetic pathway development, boosting the potential for methanol conversion into valuable compounds [174]. Nevertheless, the performance of synthetic methylotrophs falls short of those observed in native methylotrophs. For example, when comparing growth and acetate production from methanol between the most efficient synthetic methylotrophic *Escherichia coli* and *Eubacterium limosum*, it was evident that both growth and product yield were markedly lower in *E. coli* than in *E. limosum* [175]. As a different strategy, Nguyen et al. employed a comprehensive, genome-scale approach that incorporated mutagenesis, ^13^C tracer analysis, flux balance examination, and comparative transcriptomic and metabolomic studies to present the metabolism of *Methylotuvimicrobium alcaliphilum* and the mechanism behind efficient methanol consumption and formaldehyde resilience [176].

Significant advances have been made recently in synthetic methylotrophy in model organisms like *E. coli*, with the groundwork laid by pinpointing the most likely genes for methanol metabolism from methylotrophs: *mdh*, *hps*, and *phi*. Isotopic incorporation tests with ^13^C-methanol resulted in a 40% label integration into central carbon metabolites, notably hexose 6-phosphate (H6P), in *E. coli* expressing these three genes, confirming the functionality of the RuMP pathway established by Hps and Phi [177]. By physically co-localizing crucial enzymes like Mdh, Hps, and Phi into a unified complex, methanol oxidation and formaldehyde assimilation were enhanced, resulting in a 50-fold rise in methanol to F6P conversion [178]. Once these methanol assimilation pathways were set up, research efforts shifted to tackle the complexities of utilizing methanol as the sole carbon source for *E. coli* growth and energy. One significant issue is the cofactor imbalance, as methanol oxidation through Mdh is impeded when the cellular NADH to NAD^+^ ratio rises [179]. A 3.6-fold enhancement in methanol to formaldehyde conversion was achieved by linking this step to an NADH consumption cycle [178]. Alternatively, the concentration of cellular NADH was decreased by removing *maldh* that encodes NAD+-dependent malate dehydrogenase, which mimicked the strategy used by natural methylotrophs to reduce TCA cycle activity [180]. Another common strain, *S. cerevisiae*, was explored by performing ALE on laboratory strain CEN.PK, which has an uncharacterized transcriptional regulator Ygr067cp. It was found that deletion of alcohol oxidation (ADH2) and acetyl-CoA synthetase (ACS1) had severely hindered the growth on methanol [181]. On the other hand, the exact methanol assimilation mechanism in *S. cerevisiae* is still unknown. Besides a conventional host strain, a nonconventional yeast *Yarrowia lipolytica* has also been engineered for methanol utilization by introducing RuMP and XuMP pathway genes and ALE efforts [182]

##### Formate 

Formate (CHOO^−^) is a valuable biotechnology substrate because of its high solubility in water and polar solvents, a higher degree of reduction than CO_2_ and CO, and non-flammability [183]. Despite being less abundant than methanol, rapid advancements in synthesis technology, particularly in electrochemical, photochemical, and catalytic methods, promise to increase its availability. Economic efficiency is also improving, with cost predictions suggesting that formate can compete with glucose as feedstock [184].

Microbial formate assimilation employs two primary strategies naturally. The first oxidizes formate to CO_2_, extracting and reducing the power that supports carbon fixation and provides the cell with energy [185]. This process is ideally supported by formate due to its low reduction potential [186]. The known carbon-fixation pathways facilitating formatotrophic growth through complete formate oxidation include the ATP-costly CBB cycle (i.e., reductive pentose phosphate pathway) [187] and the highly ATP-efficient, albeit oxygen-sensitive, WLP (i.e., reductive acetyl-CoA pathway) [188]. Despite the latter path being energetically most efficient in utilizing formate, its application may be limited due to the product variability and anaerobic growth conditions [162,189].

The second strategy adopted by microbes to utilize formate as the only carbon source entails the fusion of formate with another intermediary metabolic product, though a portion may still undergo oxidation to furnish the cell with reduction potential and energy [190]. Formate is combined with tetrahydrofolate (THF) to promote such growth, using energy from ATP hydrolysis, resulting in formyl-THF. This compound is then transformed into methylene-THF. Methylene-THF contributes its formaldehyde component to glycine, generating serine, which changes into glycerate. Subsequently, conversions result in the regeneration of acetyl-CoA, which can be either a biomass or valuable product precursor. While the serine pathway has the capability to directly incorporate formate and oxygen insensitivity, it still consumes three ATP to produce one acetyl-CoA from one formate molecule, which causes a kinetic inefficiency [17,185].

In formate bioconversion, it has been suggested that exchanging these inefficient formate assimilation pathways with ATP-efficient alternatives could improve yield and energy efficiency. The rGly pathway was suggested as the most convenient alternative to the other ATP-infeasible and low-biomass-yielded carbon-fixation pathways [191]. One such experiment was conducted to replace the CBB cycle *Cupriavidus necator* with the reductive glycine pathway (rGly), which, despite requiring further improvements, could convert formate into valuable chemicals, thus offering a streamlined process that bypasses the costly formate separation and prevents harmful formate accumulation [192]. Recently, Sánchez-Andrea et al. [193] showed the sulfate-reducing bacterium *Desulfovibrio desulfuricans* (strain G11), which can utilize sulfate and hydrogen as energy sources, harness an autotrophic (and formatotrophic) carbon-fixation mechanism through the reductive glycine (rGly) pathway, and use formate. Its pathway coincides with the WLP route, starting from CO_2_/CO and producing 5,10-methylene-THF. Then, under the action of the glycine cleavage/synthase system (GCS), a process that includes CO_2_, NH_3_, and 5,10-methylene-THF, glycine is synthesized and undergoes further assimilation into pyruvate and biomass [9]. GCS was also introduced to *Clostridium pasteurianum* to create a non-model industrial host by heterologous expression, and anaerobic formate utilization was successfully demonstrated [194]. 

As a common industrial strain, *E. coli* was employed considerably to achieve the most optimal formate utilizer strain. The rGly pathway, one of the most promising pathway, was introduced into *E. coli* together with the serine-threonine cycle to develop a double-direction strategy, and formate was used both as an intermediate (endogenous) and as a carbon source (exogenous) [195]. Then, the same group further developed their strategy and applied all homologous and heterologous expressions possible to produce the whole glycine and serine the cell needs from formate and CO_2_ [196]. Another approach was proposed to improve obstacles in the previous work [195], by introducing the THF cycle and the reverse glycine cleavage (gcv) pathway together and to obtain a final strain that could utilize both formic acid (FA) and CO_2_ [197]. As a next step, they engineered *E. coli* by solving the NADPH generation problem by optimizing cytochrome bo3 and bd-I ubiquinol oxidase levels to acquire full growth on sole FA and CO_2_ and as high OD600 as 7.38 in 450 h [198].

Developing autotrophic organisms in formate utilization is also an important goal. For instance, Tashiro et al. used an electrochemical-biological system to reduce CO_2_ into formate in the first place and synthesized L-serine from formate using GCS-introduced *E. coli* [199]. Gleizer et al. obtained an *E. coli* strain that has CBB established to utilize formate to cover metabolic activities and uses CO_2_ as sole carbon source [73]. They employed ALE to convert a modified strain from heterotroph to full autotroph in chemostat. In another study, *E. coli* was successfully engineered to grow on formate via the rGly pathway. Further ALE boosted the biomass yield of the engineered *E. coli* strain to 2.3 g CDW/mol formate and halved the doubling time [200]. The rGly pathway was also employed in S. cerevisiae to increase tolerance (up to 500 mM) against formate by overexpression of only native enzymes [201].

It is claimed that the formate assimilation pathways of natural formatotrophs remain suboptimal for biotechnological applications and present limitations compared to industrially optimized strains, such as *S. cerevisiae* and *E. coli*, due to the costlier cultivation requirements, slower growth rates, higher sensitivity to environmental conditions, challenges in genetic manipulation due to less understood metabolic networks, and lacking optimization techniques. [14]. Despite these limitations, certain species, like *Acetobacterium woodii*, show the potential to bridge this gap. Recently, studies highlighted that *A. woodii*, when cultivated solely with formate as the carbon and energy source, demonstrated greater efficiency and speed in transforming formate to acetate than when using gaseous substrates [202,203]. This research also undertook a comparative analysis of the energy efficiencies of various acetogens and other microbes, such as formatotrophs or engineered strains capable of utilizing formate or methanol, during the growth and product formation on C_1_ or sugar substrates. The results revealed that acetogens displayed superior energy efficiency across all substrates tested, specifically C_1_ substrates, with formate demonstrating even more significant energy potential than gaseous substrates [70]. Among the acetogens, *Eubacterium limosum* emerged as promising biocatalysts for transforming formate into acetate, primarily producing acetate, during formate-fueled growth [204].

While industrially utilized microbes demonstrate enhanced yield potential and genetic manipulability than the abovementioned nonconventional organisms, cytotoxicity associated with elevated formate concentrations poses a formidable challenge [205]. The tolerance threshold for formate varies widely among organisms and is mainly dictated by formate dehydrogenase activity [118]. For instance, *Escherichia coli* encounters significant growth disruption at formate concentrations exceeding 100 mM, indicating minimal formate dehydrogenase activity [206]. Conversely, organisms such as *Saccharomyces cerevisiae*, which exhibit heightened formate dehydrogenase activity, endure and capitalize on elevated formate concentrations, underscoring the differential formate tolerance across diverse organisms [207]. Moreover, formate consumption can lead to a slight increase in medium pH, and the resulting alcohols can be toxic to certain microbes at high concentrations, potentially damaging the cell membrane and inhibiting glycolytic enzymes [208]. Therefore, strategies such as metabolic, evolutionary, and rational engineering, proven effective for other inhibitory feedstock compounds or fermentation products, could enhance microbial resilience to formate toxicity [200,209,210]. 

#### 2.2.2. Using CO_2_-Derived C_2_ Chemicals for Biomanufacturing

**Figure 5:**
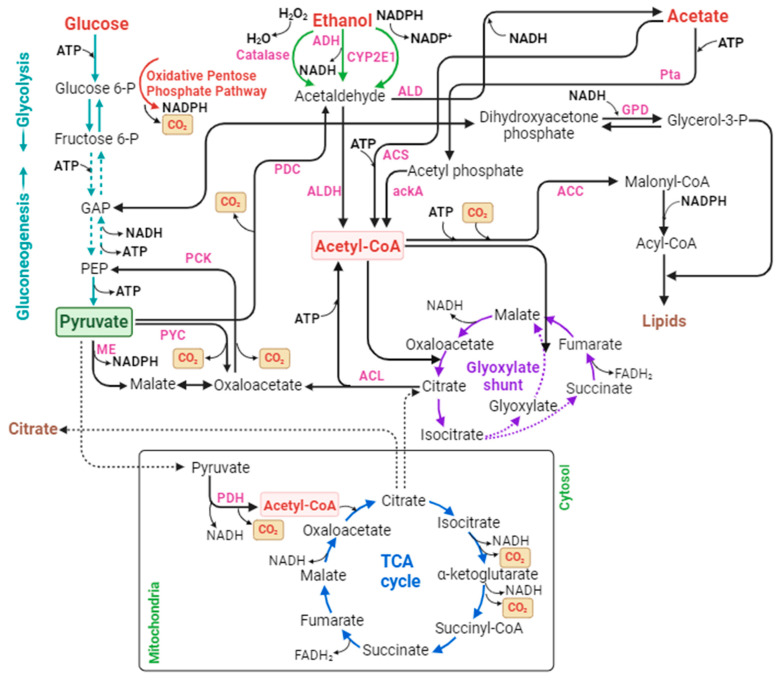
Common C_2_ chemical assimilation pathways. Metabolites: Glyceraldehyde 3-phosphate, GAP; phosphoenolpyruvate, PEP. Enzymes: acetyl-CoA carboxylase, ACC; alcohol dehydrogenase, ADH; aldehyde dehydrogenase, ALD; acetaldehyde dehydrogenase, ALDH; acetyl-CoA synthetase, ACS; cytochrome P_450_2E1, CYP2E1; ATP-citrate lyase, ACL; glycerol-3-phosphate dehydrogenase, GPD; malic enzyme, ME; pyruvate dehydrogenase complex, PDC; phosphoenolpyruvate carboxykinase, PCK; pyruvate carboxylase, PYC; pyruvate kinase, PYK. Multi-step reactions are presented by dashed arrows in related color. Black dashed arrows represent metabolite transfer. The figure was created with BioRender.

C_2_ chemicals, mainly ethanol and acetate, have garnered interest as alternative substrates for biomanufacturing, especially in the production of biofuels, pharmaceuticals, and biopolymers [211]. One of the key challenges in utilizing C_2_ chemicals as substrates in biomanufacturing is the need to expand and engineer the native metabolic pathways of microorganisms to efficiently convert these substrates into value-added products. This is achieved through various metabolic engineering strategies, such as overexpressing native or heterologous enzymes, redirecting carbon flux, and eliminating competing pathways [5]. The common C_2_ chemical assimilation pathways are shown in Figure 5.

##### Acetate

Acetate (CH_3_COO^−^) typically denotes the disassociated form of acetic acid (CH_3_COOH), holds significant economic value for biomanufacturing, and the production volume worldwide is expected to be approximately 24.5 million metric tons annually by the year 2025 [11]. Its cost ranges between $350 and 450 per ton, making it slightly more economical than traditional sugars like glucose, which cost about $500 per ton [212]. The acetate production, with around 75% of it accomplished through chemical catalysis, encompasses methods such as methanol carbonylation, ethylene oxidation, and alkane oxidation [213]. Additional methods for acetate synthesis include the hydrolysis of lignocellulosic biomass, anaerobic digestion, syngas fermentation, and microbial electrosynthesis. One of the green sides of these routes is using waste streams. For instance, lignocellulosic biomass and anaerobic digestion could leverage waste biomass and industrial or agricultural wastes as substrates. Furthermore, processes like syngas fermentation, microbial electrosynthesis, and chemical catalysis utilize CO_2_ as their primary raw material in C_1_ gas conversion [214]. This highlights the considerable potential of using acetate as potential feedstock regarding environmental friendliness and sustainability, particularly pertinent to achieving carbon neutrality.

The process of utilizing and metabolizing acetate for biochemical production starts with the transportation of acetate from the external environment into the cell, continues with the assimilation of acetate to acetyl-CoA, and, at the end, the chemicals formatted from acetyl-CoA. The acidity level within the moderately basic cellular fluid, typically with a pH value between 7.5 and 7.6, significantly exceeds the pKa value of HAc. Thus, acetic acid increases intracellular acidity to some extent by dividing into an acetate anion (Ac^−^) and a hydrogen ion (H^+^) [11]. Acetic acid can be toxic to cells, even at concentrations less than 5 g/L [213]. Other than the toxicity and proton imbalance, there are more theories explaining the inhibitory effect of acetate on cell growth. These include (i) alterations in membrane permeability and integrity; (ii) changes in amino acid metabolism, where weak acids hinder the use of specific amino acids and the production of methionine, leading to the buildup of toxic cysteine; and (iii) induced programmed cell death, where high-concentration acetate causes accumulation of reactive oxygen species and impairs energy metabolism in mitochondria, leading to chromatin and nuclear DNA denaturation and subsequent programmed cell death [215].

When microorganisms utilize acetate as the sole carbon source, it is first converted to acetyl-CoA, primarily achieved through two enzymatic routes. The first route involves the formation of an intermediate, acetyl-adenosine monophosphate (acetyl-AMP), which subsequently converts to acetyl-CoA [213]. This pathway requires two moles of ATP due to forming AMP and ADP, marking it as a more energetically expensive route. On the other hand, the acetate kinase-phosphotransacetylase (AckA-Pta) catalyzes acetate to acetyl-phosphate first and then converts it to acetyl-CoA. It represents a reversible mechanism allowing bidirectional conversion between acetate and acetyl-CoA. This pathway consumes only one mole of ATP, making it less energy-demanding than the ACS pathway. Nevertheless, the ACS pathway possesses a high affinity for acetate, around 35 times higher than the AckA-Pta pathway, thus playing a critical role in efficient acetate assimilation, particularly in low-acetate conditions. Moreover, despite its role in acetate production and consumption, the AckA-Pta pathway exhibits a lower affinity for acetate, primarily contributing to acetate production overconsumption [11].

An alternative route exists in certain bacteria, such as *Pseudomonas* sp. and acetic acid bacteria, involving the enzyme succinyl-CoA: acetate CoA-transferase (SCACT). This mechanism eliminates ATP consumption, using succinyl-CoA to convert acetate into acetyl-CoA. Therefore, the SCACT pathway is a significant supplement or alternative to the ACS and AckA-Pta pathways, especially under conditions where these two are non-functional or absent. These acetate assimilation pathways, including aerobic and anaerobic mechanisms, are widespread across several microbial species and constitute the first step of acetate metabolism [213]. Understanding these metabolic routes and their energetic requirements enriches our knowledge of microbial physiology and aids in industrial biotechnology applications by optimizing acetate metabolism in host organisms like *E. coli* [216].

Acetyl-CoA, derived from acetate, plays a pivotal role as a precursor for extending carbon length, and it is primarily incorporated into two metabolic pathways: the tricarboxylic acid (TCA) cycle or the glyoxylate shunt, leading to an increase from C_2_ to C_4_ carbon compounds. Notably, the glyoxylate shunt significantly impacts cell growth when acetate is utilized as the primary carbon source. This pathway divides isocitrate into glyoxylate and succinate via isocitrate lyase (ICL). Following this, glyoxylate is transformed into malate using another acetyl-CoA molecule [217]. Both succinate and malate are crucial to the TCA cycle, being further oxidized to form fumarate and oxaloacetate (OAA), which aid energy generation and higher carbon compound synthesis. Within the TCA cycle, the transition from isocitrate to α-ketoglutarate, facilitated by isocitrate dehydrogenase (IDH), represents an essential step that vies with ICL, thereby affecting carbon flux distribution. Both the glyoxylate shunt and the TCA cycle play a crucial role in acetate absorption due to their role in energy generation and carbon movement [218].

Nevertheless, acetate is less preferable to glucose as a carbon source when generating ATP and NADPH for most acetate-utilizing bacteria. It is worth noting that acetate yields a significantly lower energy content, with 10 ATPs per mol, compared to 38 ATPs per mol for glucose [219]. Consequently, acetate’s low-energy content might be a limiting factor for its absorption and cell growth. Given that ATP or NADPH is required for most chemical synthesis from acetate, ensuring efficient energy supplementation through metabolic engineering or other techniques is vital for acetate assimilation and biochemical production. To manufacture biochemicals effectively from acetate, certain traits are indispensable in these strains: a high level of acetate tolerance, improved activation of acetate to acetyl-CoA, enhanced acetate assimilation, and efficient chemical production [220].

In recent years, various microbes have been metabolically engineered to create biochemicals, with acetate serving as the carbon source, producing various substances, including acids, alcohols, esters, and other chemicals. In the context of C_2_-biomanufacturing, the use of acetate as a feedstock has been extensively researched, including native acetogens and genetically modified organisms that can utilize acetate. These include strains such as *E. coli* [221], *Corynebacterium glutamicum* [222], *Pseudomonas putida* [223], *S. cerevisiae* [224], *Cryptococcus curvatus* [225], *Rhodotorula glutinis* [226], *Yarrowia lipolytica* [227], and *Aspergillus oryzae* [228], among others. Moreover, over 20 value-added chemicals have been produced, with acetate as the main carbon source. Notable examples include poly(3-hydroxybutyrate) (PHB) [229], aromatic amino acids [230], lipids [231], acetate esters [232], and natural products such as isoprenoids that are derived from acetyl-CoA [233]. However, a significant challenge in using acetate is its low concentration (typically 20–30 g/L) when produced from numerous upstream waste utilization processes. Such dilute feedstock solutions further dilute when added into the microbial culture, potentially leading to a low product titer, particularly in batch operations. In response to this challenge, Xu et al. proposed an innovative approach using a continuous bioreactor with a cell recycling unit to produce triacylglycerides (TAGs), which are intracellular products that accumulate in the bioreactor with host cells such as Yarrowia lipolytica [6]. Another known strategy is ALE to increase the acetate tolerance in microorganisms. This agrees with the fact that microorganisms produce acetic acid during glucose fermentation and consume this acetic acid when carbon is limited. This consumption may be increased by adding suitable acetic acid salts to balance pH, which makes candidate organisms tolerate and consume acetate more [218].

##### Ethanol

Ethanol (C_2_H_5_OH), a simple alcoholic compound, has a broad range of applications spanning various industries, including chemical, food, medical, and health. It represents an economically viable raw material. Nonetheless, its primary sources, such as corn, are starch-based, raising concerns due to their competition with food production and considerable CO_2_ emissions during processing. The compound can be generated from renewable sources such as biomass fermentation, using sugars, starch, or cellulose as raw materials [234]. It has been established in product manufacturing of beverages, flavors, fuels, dyes, disinfectants, antifreeze, and paint [235]. Despite its versatile utilization, the exploration of ethanol as a metabolic engineering feedstock is not yet thoroughly investigated [236].

The conversion of ethanol into productive biochemical pathways typically ensues through two main mechanisms. The initial route involves the enzymatic action of alcohol dehydrogenase and acetaldehyde dehydrogenase, transforming ethanol into acetaldehyde and subsequently into acetyl-CoA. Alternatively, a route more prevalent in eukaryotes, such as *S. cerevisiae*, initiates the transformation of ethanol to acetate using acetaldehyde as an intermediary, which is then integrated into acetyl-CoA. Microbial species like *Clostridium acetobutylicum* and *E. coli* predominantly utilize the former pathway, whereas in *S. cerevisiae*, the oxidation of ethanol to acetaldehyde is facilitated by alcohol dehydrogenase enzymes, specifically Adh2 or Adh4. This acetaldehyde is then converted to acetate via aldehyde dehydrogenase enzymes, namely Ald4 and Ald5. These processes generate NADH, which is crucial for ATP regeneration, thus providing a higher theoretical yield from ethanol than acetate for reducing product production. However, a significant caveat is that ethanol assimilation can be heat-intensive and oxygen-dependent, potentially amplifying the overall production expenditure [9,237].

In some synthetic hosts like *E. coli*, metabolic engineering has been deployed to optimize ethanol assimilation. This includes the manipulation of the acetaldehyde dehydrogenase and alcohol dehydrogenase enzymes for efficient ethanol growth [238]. These engineered strains can be further refined to produce valuable products like polyhydroxy butyrate (PHB) or prenol from ethanol [229]. Ethanol can also be utilized as the sole or co-substrate with glucose to produce valuable compounds like the artemisinin precursor in *S. cerevisiae* [239]. Further strategies have explored the genetic modification of *E. coli* strains for ethanol utilization. These modifications often introduce ethanol catabolism pathways into *E. coli*, such as those found in *Aspergillus nidulans* [240]. By expressing different alcohol dehydrogenases and aldehyde dehydrogenases in *E. coli*, there is a potential for efficient ethanol utilization and production of value-added products from ethanol [238]. One example is introducing a two-step ethanol utilization pathway (EUP) into *E. coli*, which has shown promising results in generating polyhydroxy butyrate (PHB), an acetyl-CoA-derived product [236]. The engineered *E. coli* strain demonstrated robust growth on ethanol as the sole carbon source. It produced 1.1 g/L of PHB from 10 g/L of ethanol in 96 h with supplementation of a small number of amino acids. To expand the range of potential acetyl-CoA-derived compounds from ethanol, this EUP was coupled with a prenol biosynthetic pathway. The resulting strain produced 24 mg/L of prenol from a medium containing ten g/L of ethanol in 48 h. As an exciting new approach, C2-biomanufacturing using ethanol as the sole carbon source has opened the possibility of producing acetyl-CoA-derived chemicals. Significantly, this strategy has led to a higher theoretical yield for producing acetyl-CoA-derived chemicals from ethanol than other sources. For example, the PHB yield from ethanol was 2-fold higher than that from acetate [229]. Further technological developments and metabolic engineering strategies will likely enhance these processes, making CO_2_-derived ethanol an abundant, renewable, and affordable substrate to fuel ethanol-based fermentation processes [241].

Taken together, both C_1_ and C_2_ chemicals derived from the electrochemical fixation of CO_2_ can serve as carbon and energy sources for further biomanufacturing with various microorganisms. The major biochemical reactions to generate ATP from the most common C_1_/C_2_ substrates are summarized in Table 2, which may potentially provide guidance for further pathway design and bioconversion yield predictions in future. 

#### 2.2.3. Biomanufacturing with Syngas via Gas Fermentation

In addition to the CO_2_-derived liquid C_1_/C_2_ chemicals that can be used as the alternative feedstock for biomanufacturing of fuels and chemicals, synthesis gas, or syngas, which consists of carbon monoxide (CO), hydrogen (H_2_), carbon dioxide (CO_2_), nitrogen (N_2_), and some higher hydrocarbons, can also be used as an economical feedstock option. The percentage of CO in syngas can range between 5 and 60%, and the gas can be steam reformed to enrich the H_2_ content [242]. CO can be obtained from CO_2_ via electrochemical conversion and H_2_ can be produced as a product of electrolysis process with water. Syngas can also be produced from biomass gasification, an endothermic process that occurs at temperatures of 750–800 °C and utilizes materials like lignocellulosic biomass and municipal solid waste as feedstocks [243,244]. Despite its promise, the process has some drawbacks. It requires a considerable input of heat energy, and the feedstock must maintain a degree of homogeneity for efficient operation [245]. Heterogeneous feedstocks can lead to wide variations in product composition, necessitating pre-treatment and post-treatment steps that can escalate operational costs [246]. Conversely, the thermochemical process involves gasifying carbonaceous materials into syngas and converting it into biofuels [247]. Syngas can be converted to diesel, methanol, or ethanol using the Fischer–Tropsch (FT) process, which uses chemical catalysts. Still, this method requires high temperatures and pressures, making it less feasible [246]. Another option is using microbial catalysts to convert syngas into a variety of products, like alcohols and carboxylic acids, at milder conditions [248,249].

However, each of these platforms presents unique advantages and disadvantages. Biochemical conversion, for example, struggles with high production costs and energy demands. On the other hand, the thermochemical conversion process, while capable of utilizing all biomass components (including lignin), faces challenges like gas–liquid mass transfer limitation, low productivity, and elevated production costs [250]. Combining the two conversion processes, such as electrochemical conversion CO_2_ into CO, biomass gasification, and syngas fermentation, could be a solution to these problems. Syngas fermentation, compared to Fischer–Tropsch Synthesis (FTS), is seen as a superior option due to its operational flexibility, end-product variety, and cost-effectiveness. This technology could serve as a sustainable way of supplying feedstock for fermentation. Integrating gasification with syngas fermentation could bring together the benefits of thermochemical (full conversion of lignocellulosic biomass) and biochemical (flexibility in CO/H_2_ ratio of the substrate and end products) technologies, mitigating the complexity of pre-treatment steps and the high enzyme and operational costs of biomass valorization [251]. This approach has the potential to be directly implemented in industries that release high levels of exhaust gases, like steel manufacturing, oil refining, and petrochemistry.

However, syngas fermentation processes still have challenges to overcome, such as bacterial biomass washout, low gas solubility, and limited mass transfer rates at the gas–liquid interface. These challenges demand further research and innovation to boost microbial activity or limit the exposure of microorganisms to excessive shear stress, ultimately reducing operational costs [252]. Microbial conversion of CO, H_2_, and CO_2_ to acids and alcohols via acetogenic bacteria operates via the reductive acetyl-CoA or WLP, as mentioned in earlier sections. These biological methods offer several advantages such as high tolerance to trace contaminants, high product specificity, and being sustainable, environmentally friendly, and cost-effective [253]. Despite these obstacles, gas fermentation offers a promising route for sustainable fuel production and waste recycling. It provides feedstock flexibility, non-food biomass utilization, and total carbon utilization, including lignin from woody biomass, offering significant advantages over sugar fermentation. Moreover, if the process limitations can be overcome, gas fermentation could provide a more selective, robust, flexible, and cost-effective option than the thermocatalytic Fischer–Tropsch synthesis, suggesting it is a promising technology for mitigating global warming and fulfilling increased liquid fuel demand, especially in transportation [254].

#### 2.2.4. Current Attempts to Industrialize Microbial CO_2_ Fixation

The dream of establishing a CO_2_-based biorefinery is a long-standing challenge. The rise in CO_2_, primarily due to anthropogenic activities, has significant ecological impacts. There is a pressing demand to develop technologies for sustainable capture and utilization of CO_2_. In this regard, renewable energy generation and usage have garnered significant interest in achieving a carbon-neutral environment. Microbial fermentation is one of the best ways to reach this aim, and the use of CO_2_-based feedstocks as substrates has been extensively explored to produce various valuable products. These include food ingredients like alternative proteins, lipids, starch, nutraceuticals, specialty chemicals such as flavors and fragrances, pharmaceuticals, agrochemicals like plant hormones, and bioenergy sources, including fuels and hydrogen [255]. Various methods like biological CO_2_ conversion using microbes, chemo-catalytic CO_2_ conversion via organic or inorganic catalysts, light-induced or electrocatalytic CO_2_ conversion, and catalytic hydrogenation of CO_2_ have demonstrated the capability to convert CO_2_ into bio-based products [256]. However, from a large-scale perspective, none of these methods can merely resolve CO_2_ capture and usage problems.

LanzaTech has successfully deployed gas fermentation technology to produce carbon-intelligent products ranging from monomeric and polymeric materials to fragrances, solvents, chemicals, and fuels [257]. They produce substrates like acetone, ethanol, and lactate from waste syngas and flue gas using acetogens and autotrophic bacteria, where CO_2_, CO, and H_2_ serve as carbon and reducing energy sources [254]. LanzaTech’s partnerships include major industrial players like Shougang Group’s Jingtang Steel Mill, Arcelor Mittal Steel Company, Indian Oil Corporation, Tata Steel Europe, and more, demonstrating its global reach and impact [258]. Additionally, LanzaTech’s work extends to converting CO_2_ to acetone and isopropanol at an industrial pilot scale [71] and producing starch in the form of amylose and amylopectin in a cell-free system [53]. Similarly, the Siemens Energy and Evonik partnership established the world’s first fully automated CO_2_ electrolyzer in 2020, producing syngas to make butanol and hexanol with *Clostridium* strain in a 2000 L bioreactor. This project aims to produce 10,000 tons of butanol annually using 25,000 tons of CO_2_ [259].

High-profile CO_2_ capture projects have been developed in Italy, Germany, New Zealand, the Netherlands, the United Kingdom, Canada, China, and the USA. These initiatives underscore the global effort to harness CO_2_ for sustainable industrial applications [23]. Numerous start-up companies including Air Protein Inc. [260], Deep Branch Biotechnology Ltd. [261], Kiverdi [262], Solar Foods [263], and NovoNutrients [264], are notable in biotechnological CO_2_ utilization for producing protein and food ingredients. The success of these ventures hinges on various factors, including the cost of hydrogen, feedstock availability, market size, and growth rates. Continuous technological development, economies of scale, supportive policies, and market incentives, are crucial for advancing biotechnological utilization and valorization of CO_2_.

## 3. Challenges and Future Perspectives

### 3.1. Challenges for Biomanufacturing with Direct Fixation of CO_2_

The conversion of inorganic carbon (CO_2_) into organic compounds offers a promising strategy to mitigate the greenhouse effect and furnish sustainable resources. This method has potential implications for addressing climate change and utilizing CO_2_ as an economical substrate for producing fuels, chemicals, food ingredients, pharmaceuticals, and industrial materials. The rapid advances in chemical, electrochemical, and biotechnological research methods and tools indicate the imminent identification of novel carbon-fixing enzymes and pathways, which makes it feasible for directly fixing and converting CO_2_ into desired fuels or chemical products. However, despite these discoveries, the current natural or engineered carbon-fixation systems are plagued by inefficiencies and a lack of adaptability for genetic modifications, making them inadequate for industrial applications. There are several major challenges to be addressed before the one-step or direct fixation of CO_2_ strategy can be applied in large-scale applications for high-yield production of fuels and chemicals from CO_2_:

(1) Only low-energy utilization efficiency can be achieved when light is used as the energy source to fix CO_2_. Green plants, algae, and certain bacteria are capable of using sunlight via the photosynthesis process to capture and fix CO_2_ into carbohydrates, but at low-energy efficiency, with less than 1% of the sunlight energy stored in the biosynthesized chemicals [5,265].
(2) Energy-intensive chemicals such as H_2_ gas can be used to fix CO_2_ and provide the reducing power to convert CO_2_ into the desired carbohydrate products, but there are concerns of extra material cost, technical challenges of using gas for fermentation, increased process complexity, and operating safety due to the use of H_2_ gas or similar energy-intensive materials.
(3) A very limited number of microbial hosts, genetic manipulation methods and tools, and pathway engineering strategies are available for more generalized applications of direct CO_2_ fixation and conversion. Many synthetic pathways for direct CO_2_ fixation face major challenges, such as enzymes with toxicity to host cells or with non-compatible optimum temperatures. Innovations such as the allyl-CoA carboxylase/reductase, which boasts an activity rate 37 times that of the CBB cycle, show promise in addressing this [26]. Introducing mechanisms to concentrate carbon also seems to be a viable strategy to enhance the carbon flux in these pathways. With synthetic biology’s progress, exploring and designing novel pathways might be the key. Predictions even suggest that certain pathways, like those using phosphoenolpyruvate carboxylase, could potentially offer two to three times the carbon-fixation rate of the Calvin cycle [56].
(4) Microbial electrosynthesis (MES) can be used to produce certain fuels or valuable organic acids [92,93,94] by utilizing a biofilm on an electrode as a catalyst to directly reduce CO_2_ to the products [23], but the species of the microorganisms and the categories of the fuels and chemicals that can be produced are very limited. Acetate is the current major product and its production titer and yield are still too low, which significantly increases the downstream recovery cost [266]. In addition, there is strict requirement for the materials that can be used for cathode. More challenges for further process design and scale-up are expected for large-scale applications in future [266].

### 3.2. Challenges for Biomanufacturing with CO_2_-Derived C_1_/C_2_ Chemicals

Due to the overall low-energy efficiency and/or product yield from the biomanufacturing process with one-step/direct CO_2_ fixation, the two-step CO_2_ fixation and conversion strategy is considered more promising for future biomanufacturing of various fuels and chemicals, which uses C_1_/C_2_ substrates derived from CO_2_ via electrochemical catalysis. However, there are also several major challenges need to be addressed:

(1) Mass transfer challenges limits the microbial fermentation productivity when the CO_2_-derived C_1_ gases, such as CO or CH_4_, are used as the substrate. Metabolic engineering strategies for using appropriate microorganisms to metabolize the C_1_ gases are also to be established and further optimized. In addition, safety concerns are also another challenge that may limit the use of CO for biomanufacturing.
(2) Though formic acid and acetic acid can be used as the substrate for biomanufacturing, most current electrochemical catalysis processes can only fix CO_2_ into the form of formate or acetate salts in aqueous solution, which need to be further treated with acid and base and go through a complicated purification process to obtain the acid products so that they can be fed into the bioreactor for microbial fermentation. Progress has been achieved in electrochemically fixing CO_2_ into nearly pure formic acid [267], but the productivity needs to be further improved for large-scale application. Comparing to the electrochemical reduction in CO_2_ into formic acid, converting CO_2_ into acetic acid at high yield is still a challenge [268].
(3) Direct feeding too much formic acid or acetic acid into a bioreactor may cause sudden acidic pH spikes in fermentation and kill the microbial cells. Therefore, new formic/acetic acid feeding strategies should be developed to avoid/minimize pH spikes in a bioreactor while providing enough substrate(s) for cell growth and product formation [269,270].
(4) Methanol and ethanol can be used as fermentation substrates with high-energy densities, but high concentrations of the alcohol substrates may cause toxicity to the microbial cells. In addition, further metabolic engineering strategies for efficient assimilation of methanol and/or ethanol should be explored for significantly higher product yield.

### 3.3. Future Perspectives for Biomanufacturing with CO_2_

**Figure 6:**
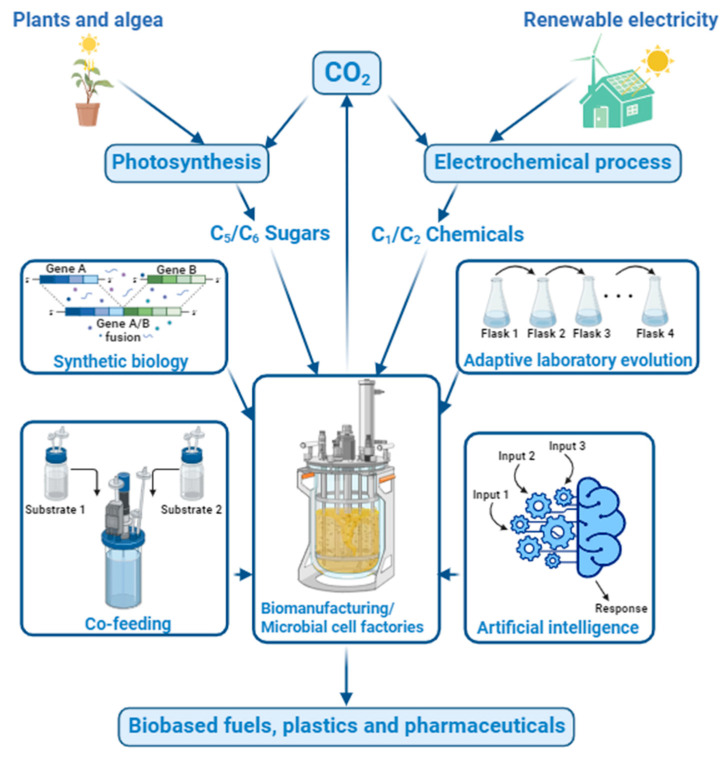
A brief summary of various methods for using CO_2_ as feedstock for biomanufacturing, which shows the major promising approaches to efficiently fix CO_2_ and convert it into desired products through a carbon-neutral or -negative biomanufacturing process [162,271]. The figure was created with BioRender.

**Figure 7:**
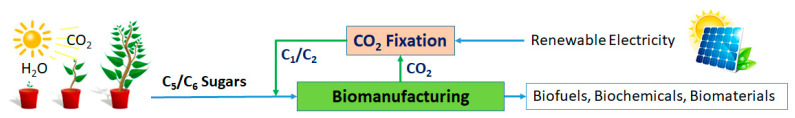
A conceptual diagram for a novel biomanufacturing platform that can produce fuels and chemicals from C_5_/C_6_ sugars at zero or near zero life cycle carbon emissions via in situ CO_2_ recycling.

The overuse of fossil oil-based or -derived fuels, chemicals, and materials has led to increased carbon emissions, which are one of the major contributors to global climate change. Biomanufacturing with renewable or waste feedstocks is considered as a promising and sustainable route to replace the current petrochemical methods for producing all fuels, chemicals, and materials that are needed in our daily life. Feedstock or raw materials, typically obtained from land-based biomass in the format of starch, sugars, and fats, contribute to a significant portion of the biomanufacturing product cost. Using CO_2_ or CO_2_-derived chemicals as biomanufacturing feedstock not only reduces the material cost, but also contributes to the global effort in reducing carbon emissions and achieving the carbon-neutral or -negative goal. While significant progresses have been achieved to demonstrate the feasibility of using one-step or two-step strategies for biomanufacturing with CO_2_, major challenges and technical barriers still exist, as described earlier. Figure 6 shows a brief summary of using various methods that have been developed or will be developed for using CO_2_ as feedstock for biomanufacturing. The following research efforts and perspectives will be expected in future:

(1) Using advanced synthetic biology to create new microbial cell factories to utilize CO_2_ and CO_2_-derived chemicals for high-yield biomanufacturing: Researchers are now at the forefront of devising more efficient synthetic systems. This involves engineering pivotal enzymes and transferring whole or partial carbon-fixation pathways into heterotrophic cells, enabling them to perform carbon fixation. A testament to these efforts includes the creation of pathways like the MCG pathway and the CETCH cycle using different carboxylases [26]. Although the enhancement in carbon-fixation rate remains modest, these innovations may lead to designing more adept systems. Host selection also is a challenge for keeping CO_2_ fixation sustainable. For example, most CO_2_-fixing microbes cannot tolerate high CO_2_ concentrations, necessitating research into strains that can endure and efficiently process higher levels of CO_2_ or CO_2_-derived substrates. Adaptive laboratory evolution (ALE) methods may be applied to help develop more robust production strains that are suitable for large-scale applications.
(2) Using artificial intelligence (AI) to guide the discoveries of new strains, metabolic pathways, enzymes, and fermentation process controls that may lead to complete bioconversion of CO_2_ or CO_2_-derived substrates [271,272,273]: This may also help discover new valuable products that may be produced from the pathways using CO_2_ or having CO_2_ as the major intermediates. More advanced process, such as continuous biomanufacturing with extremely high yield and productivity, can also be developed [8].
(3) Exploring a cofeeding strategy that uses a mixed C_1_ and C_2_ substrates for biomanufacturing: Current electrochemical reduction in CO_2_ focuses on maximizing the production of a single C_1_/C_2_ product at high yield and selectivity. However, the microbial cells may be capable of using a mixed C_1_ and C_2_ feed for producing a desired fermentation product. This may help relieve the burden in the electrochemical catalysis system and significantly reduce its cost. More strain engineering and fermentation process development work should be conducted to use a medium or feed with mixed C_1_/C_2_ substrates, including methanol, formic acid, ethanol, and acetic acid, for various biomanufacturing purposes. A joint research effort between the electrochemists, biologists, and chemical engineers are expected to achieve the goal.
(4) Developing an advanced process control strategy based on online monitoring/measurements of dissolved CO_2_ in an aqueous medium, exhausted CO_2_ in off-gas flow, and the cellular redox levels: Technologies for measuring dissolved CO_2_ in liquid and gas-phase CO_2_ have been well established and become commercially available. Monitoring redox cofactor (NAD/NADH, NADP/NADPH, FAD/FADH_2_) balance has also been investigated and demonstrated a capability for advanced fermentation control to further improve the biomanufacturing yield [274,275,276]. In particular, a nutrient-induced metabolic shift for high productivity and low-waste generation has been demonstrated in cultures of various cell lines and products. However, as the cells rapidly respond to culture conditions, it is crucial to closely monitor their metabolism for a controlled balance between the target metabolic pathway and unfavorable consequences. In particular, during biosynthesis of bioproducts from CO_2_-derived C_1_/C_2_ substrates, additional reduction power (NADH, NADPH, FADH_2_) has to be supplied to produce compounds whose degree of reduction is higher than that of the substrate [277,278]. Therefore, adjusting the metabolic status and pathways for improved NADH/NADPH in microbial cells is an effective method to enhance the biosynthesis of many bioproducts [277,279,280]. Moreover, other parameters like temperature (to consider O_2_ and CO_2_ solubility), pH (regarding the host optimal pH), dissolved oxygen, and total inorganic carbon should be optimized for reaching higher yields [281,282].
(5) Developing a novel biomanufacturing platform that can produce fuels and chemicals from sugars at zero or near-zero life cycle carbon emissions via in situ CO_2_ recycling: Most microbial fermentation processes that use C_5_/C_6_ sugars as substrates have nearly 50% or more carbon loss due to the need for metabolizing a portion of the sugar substrate into CO_2_ to generate energy (ATP) and cofactors for cell growth and biosynthesis. To date, there has been very rare research aiming for biomanufacturing with direct recycling of the exhausted CO_2_. The capturing and fixation of CO_2_ into C_1_/C_2_ chemicals can be achieved via similar electrochemical catalysis processes [116,283]. There are several trials to combine electrochemical reduction in CO_2_ and the fermentation of its reduced products. However, there is still a long way to go for the optimization of this combined system to work effectively [284]. The developed new biomanufacturing platform should employ newly engineered strains that can co-utilize C_5_/C_6_ sugars and CO_2_-derived C_1_/C_2_ chemicals for producing the desired fermentation products as shown in Figure 7. Recycling the exhausted CO_2_ back to fermentation not only avoids/minimizes the CO_2_ release from the biomanufacturing processes, but also maximizes the use of the renewable feedstocks for significantly higher product yield.

## 4. Conclusions

This review summarized the most recent advancements and strategies in CO_2_ fixation and conversion into industrially valuable chemicals. The path to efficient CO_2_ fixation is fraught with challenges, ranging from biological to technical. Nonetheless, the rapid advancements in synthetic biology and multi-disciplinary collaborations offer a promising future for the field. Addressing these challenges will provide avenues for sustainable resource generation and significantly contribute to climate change mitigation. Continued research and innovation are vital to bringing these promising laboratory-level techniques to commercial reality and industrially available candidates in addressing GHG emissions.

## Figures and Tables

**Table 1:** General strategies for biotechnological fixation of CO_2_.

Methods	Major Steps and Overall Reaction of CO_2_ Fixation	
One-step/Direct CO_2_ fixation and conversion	Calvin–Benson–Bassham (CBB) Cycle: 3CO_2_ + 12 ATP → GAP (→ ½ Glucose) Wood–Ljungdahl Pathway (WLP): 2CO_2_ + CoA + 4H^+^ + 4e^−^ → Acetyl-CoA + 2H_2_O Reductive Glycine Pathway (rGlyP): 3CO_2_ + 3H_2_ → Pyruvate Reductive Tricarboxylic Acid Cycle (rTCA): 2CO_2_ + CoA + 2ATP → Acetyl-CoA 3-Hydroxypropionate (3HP) Bicycle: 2CO_2_ + 2ATP → Glyoxylate; CO_2_ + Glyoxylate + ATP → Pyruvate 3-Hydroxypropionate/4-Hydroxybutyrate (HP/HB) Cycle: 2CO_2_ (HCO_3_^−^) + CoA + 4ATP → Acetyl-CoA Dicarboxylate/4-Hydroxybutyrate (DC/HB) Cycle: 2CO_2_ (HCO_3_^−^) + CoA + 3ATP → Acetyl-CoA	
Two-step CO_2_ fixation and conversion	Step 1 (electrochemical catalysis): CO_2_ + H_2_O + electricity → C_1_/C_2_ chemicals	Step 2 (biomanufacturing): C_1_/C_2_ → biofuels and chemicals
	CO_2_ + 2H_2_O + electricity → CH_3_OH + 1.5O_2_ CO_2_ + H_2_O + electricity → HCOOH + 0.5O_2_ 2CO_2_ + 3H_2_O + electricity → C_2_H_5_OH + 3O_2_ 2CO_2_ + 2H_2_O + electricity → CH_3_COOH + 2O_2_ CO_2_ + electricity → CO + 0.5O_2_ CO_2_ + 2H_2_O + electricity → CH_4_ +2O_2_	Direct use of C_1_/C_2_: C_1_/C_2_ → fuels/chemicals + biomass Cofeeding C_1_/C_2_ and C_5_/C_6_ sugars: C_1_/C_2_ + C_5_/C_6_ sugars → fuels/chemicals + biomass

**Table 2:** ATP balance for the most common C_1_ and C_2_ chemicals, calculated regarding Figure 4 and Figure 5.

Substrate	Key Enzyme	Major Biochemical Reactions			Eq. ATP/Substrate
		Reaction 1	Reaction 2	Reaction 3	
CO_2_	N/A	CO_2_ + RuBP + 2NADPH + 2ADP + 2Pi → 2GAP + 2NADP + 2ATP	N/A	N/A	3.3
CO	N/A	CO + 5-Methyl-THF → AcCoA	N/A	N/A	6.0
Methane (CH_4_)	N/A	CH_4_ + O_2_ + NADH → HCHO + NAD	HCHO + Xu5P + ATP → 2GAP + ADP + Pi	N/A	8.7
Methanol (CH_3_OH or MeOH)	RuMp	MeOH + NADH → HCHO + NAD	HCHO + Ru5P + ATP → 2GAP + ADP + Pi	2GAP + 8ADP + Pi + 8NAD → 2AcCoA + 8ATP + 8NADH + 2CO_2_	8.7
	XuMp		HCHO + Xu5P + ATP → 2GAP + ADP + Pi	N/A	9.2
	Serine		HCHO + FAD + 3ATP + 2NADPH + 2NADH + Glycine + CO_2_ → AcCoA + FADH_2_ + 3ADP + 2NADP + 2NAD + Glyoxylate	N/A	−6.0
Formate (HCOOH)	CBB	HCOOH + NAD → CO_2_ + NADH	RuBP + CO_2_ + 2NADPH + 2ADP + Pi → 2GAP + 2NADP + 2ATP	2GAP + 8ADP + Pi + 8NAD → 2AcCoA + 8ATP + 8NADH + 2CO_2_	9.2
		HCOOH + ATP → 10-Formyl-THF + ADP + Pi	10-Formyl-THF + NADPH + NADH + CO_2_ + FADH_2_ → AcCoA + NADP + NAD + FAD	N/A	5.0
Acetate (CH_3_COOH or OAc)	Pta/ackA	OAc + ATP → ADP + AcP	AcP + CoA → AcCoA + pi	N/A	11
	ACS	OAc + ATP + CoA → AcCoA + AMP + PPi	N/A	N/A	11
Ethanol (CH_3_CH_2_OH or EtOH)	CYP2E1	EtOH + NADPH + H + O_2_ → MeCHO + NADP + H_2_O	MeCHO + NADH → NAD + OAc	OAc + ATP → ADP + AcP; AcP + CoA → AcCoA + pi	5
				OAc + ATP + CoA → AcCoA + AMP + PPi	5
	ADH	EtOH + NAD → MeCHO + NADH		OAc + ATP → ADP + AcP; AcP + CoA → AcCoA + pi	11
				OAc + ATP + CoA → AcCoA + AMP + Ppi	11
	Catalase	EtOH + H_2_O_2_ → MeCHO + H_2_O		OAc + ATP → ADP + AcP; AcP + CoA → AcCoA + pi	8
				OAc + ATP + CoA → AcCoA + AMP + PPi	8
